# Detrimental effects of flame retardant, PBB153, exposure on sperm and future generations

**DOI:** 10.1038/s41598-020-65593-x

**Published:** 2020-05-22

**Authors:** Katherine Watkins Greeson, Kristen L. Fowler, Paige M. Estave, S. Kate Thompson, Chelsea Wagner, R. Clayton Edenfield, Krista M. Symosko, Alyse N. Steves, Elizabeth M. Marder, Metrecia L. Terrell, Hillary Barton, Michael Koval, Michele Marcus, Charles A. Easley

**Affiliations:** 1 Department of Environmental Health Science, College of Public Health, University of Georgia, Athens, GA USA; 2 Regenerative Bioscience Center, University of Georgia, Athens, GA USA; 3 Department of Obstetrics, Gynecology and Reproductive Sciences, McGovern Medical School at The University of Texas Health Science Center at Houston, Houston, TX USA; 4 Neuropharmacology and Neurologic Diseases, Yerkes National Primate Research Center, Atlanta, GA USA; 5 Department of Epidemiology, Rollins School of Public Health, Emory University, Atlanta, GA USA; 6 Division of Pulmonary, Allergy, Critical Care and Sleep Medicine, Department of Cell Biology, Emory University School of Medicine, Atlanta, Georgia USA

**Keywords:** Spermatogenesis, DNA methylation, Stem-cell differentiation

## Abstract

In 1973, the Velsicol Chemical Company, which manufactured FireMaster, a brominated flame retardant, and NutriMaster, a nutritional supplement, mistakenly shipped hundreds of pounds of FireMaster to grain mills around Michigan where it was incorporated into animal feed and then into the food chain across the state. An estimated 6.5 million Michigan residents consumed polybrominated biphenyl (PBB)-laced animal products leading to one of the largest agricultural accidents in U.S. history. To date, there have been no studies investigating the effects of PBB on epigenetic regulation in sperm, which could explain some of the endocrine-related health effects observed among children of PBB-exposed parents. Fusing epidemiological approaches with a novel *in vitro* model of human spermatogenesis, we demonstrate that exposure to PBB153, the primary component of FireMaster, alters the epigenome in human spermatogenic cells. Using our novel stem cell-based spermatogenesis model, we show that PBB153 exposure decreases DNA methylation at regulatory elements controlling imprinted genes. Furthermore, PBB153 affects DNA methylation by reducing *de novo* DNA methyltransferase activity at increasing PBB153 concentrations as well as reducing maintenance DNA methyltransferase activity at the lowest tested PBB153 concentration. Additionally, PBB153 exposure alters the expression of genes critical to proper human development. Taken together, these results suggest that PBB153 exposure alters the epigenome by disrupting methyltransferase activity leading to defects in imprint establishment causing altered gene expression, which could contribute to health concerns in the children of men exposed to PBB153. While this chemical is toxic to those directly exposed, the results from this study indicate that the epigenetic repercussions may be detrimental to future generations. Above all, this model may be expanded to model a multitude of environmental exposures to elucidate the effect of various chemicals on germline epigenetics and how paternal exposure may impact the health of future generations.

## Introduction

In recent years, a major focus of research has been the Developmental Origins of Health and Disease (DOHaD)^1^. A component of DOHaD research examines the impact of pre- and post-conception risk factors on long-term health and disease progression in adulthood. In terms of pre-conception risk factors, many researchers report that toxicants and environmental exposures can potentially alter the epigenome in gametes, giving these exposures the potential to ultimately impact the health of the next generation^2–5^. In 1973, farms across Michigan mistakenly received cattle feed mixed with FireMaster BP-6, a chemical flame retardant composed of a mixture of polybrominated biphenyl compounds (PBBs), rather than NutriMaster, a food additive that increased milk production in farm animals^6–9^. PBB-laced animal products were consumed by millions of residents, and the decline in animal health, including reproductive issues, was attributed to consumption of this flame retardant^9–12^. In 1976, the Michigan Department of Public Health, now the Michigan Department of Health and Human Services, enrolled approximately 5,000 people including farmers whose farms were quarantined, workers from the Vesicol Chemical Company (exposed by manufacturing and handling the flame retardant) and others exposed to PBBs in the Michigan Long-Term PBB Study which has since been renamed the Michigan PBB Registry to study short and long-term effects of PBB on human health, with prominent interest in biological persistence, maternal transmission, chronic disease, reproductive outcomes and hormonal dysfunction^13,14^.

An early epidemiological study on the Michigan PBB Registry reported a higher prevalence of acne, skin darkening, nail discoloration, slowed healing, nausea, headache, blurred vision, dizziness, depression, fatigue, anxiety, weakness, paresthesia, loss of balance, joint, back, and leg pain, dry skin and increased sweating among various groups of exposed Michigan residents compared to unexposed dairy farmers from Wisconsin. Additionally, symptoms related to the nervous system were associated with elevated serum alanine transaminase (ALT) levels (referred to as “SGPT values” in print), suggesting an impact of PBB on the liver^6^. Highly exposed members of the cohort also appear to have increased risk of lymphomas and cancers of the digestive tract, including esophagus, stomach, liver, and pancreas, as well as breast cancer^15–17^. Exposure to PBBs in this cohort has been linked to increased odds of thyroid disease^18–20^, although one study found conflicting results^21^. Interestingly, the children of highly exposed mothers appear to have higher rates of reproductive problems compared to children of women with lower exposures with adult sons having a three-times higher likelihood of reporting development of genitourinary conditions^22^ and daughters having a four-times higher odds ratio for spontaneous abortions^23^ in addition to developing pubic hair and menarche over a year earlier compared to unexposed girls^24^. For the women who were exposed as adults, an increased risk of spontaneous abortions was not evident in association with PBB exposure^25^, but other reproductive outcomes were not systematically evaluated in this first generation of the cohort.

In a review, Curtis, *et al*.^13^ summarized studies showing that the structures of PBBs are similar enough to chemicals associated with more unanimous and widespread environmental exposures, such as polychlorinated biphenyls (PCBs) and polybrominated diphenyl ethers (PBDEs) that they likely act in similar ways in the body. PBBs, PCBs, and PBDEs not only act as endocrine disruptors but also may alter the epigenome in ways which could explain diseases which are both associated with changes in the epigenome and exposure to each family of chemicals, including PBBs. Those enrolled in the PBB Registry have a variety of health concerns that align with diseases associated with epigenetic alterations, such as cancers and reproductive issues^26–28^. Curtis, *et al*.^19^ reported that in peripheral blood, PBB exposure was associated with a depletion of methylated CpGs in promoter and heterochromatic regions and enrichment in enhancer and insulator regions of DNA. These results also indicated that CpG methylation changes associated with PBB exposure were near binding sites for AHR pathway and estrogen receptor transcription factors. Though these findings were compelling for explaining the health effects observed in those directly exposed to PBBs, the question of why the children of those directly exposed had different, sometimes more severe health effects remained. Here, we demonstrate the ability to fuse epidemiological and epigenetic data with a stem cell model of human spermatogenesis to understand how exposure to a specific environmental toxicant can impact the epigenome in human sperm and potentially contribute to reproductive, developmental and endocrine issues in offspring.

Given the observation of different health effects observed in the children of the PBB Registry compared to their parents, we hypothesize that beyond impacting the epigenome of somatic cells, exposure to PBBs may also alter the heritable epigenome to cause intergenerational health effects not seen in generations directly exposed. To study this effect, we focused on the men of this cohort whose exposure would not lead to *in utero* or breastfeeding exposure. Further, since these reproductive defects could not be attributed to abnormal sperm counts or sperm morphology, as PBB-exposed men do not show declines in semen parameters^29^, we hypothesize that PBB153, the primary component of FireMaster BP-6^7,9^, disrupts the epigenetic regulation during spermatogenesis, specifically the establishment of parent-of-origin imprints.

### PBB153 induces epigenetic alterations in male gametes

**Figure 1:**
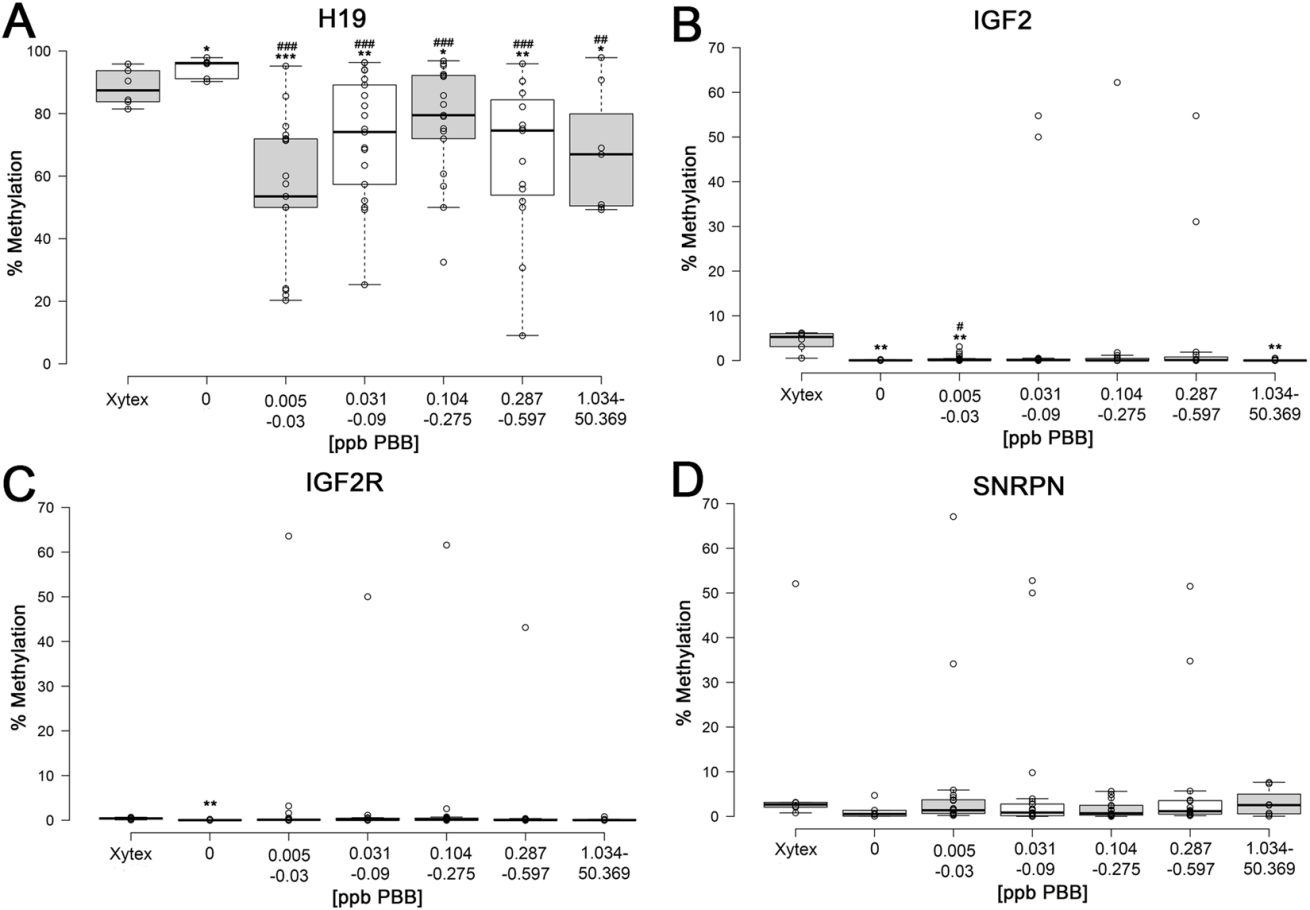
PBB153 serum level correlates with decreased methylation at the ICR of a paternally silenced gene in sperm. (**A–D**). Percent methylation of *H19* (paternally silenced), *IGF2* (differentially methylated region, hypomethylated in sperm), *SNRPN* (maternally silenced), and *IGF2R* (biallelically expressed) ICRs. PBB Registry donors with circulating PBB levels have decreased percent ICR methylation at the *H19* loci. Xytex refers to control samples purchased from Xytex Cryo International. Samples were compared to both Xytex control (significance in one-sided-independent *t-*test designated by asterisks where *is p < 0.05; **is p < 0.01; and ***is p < 0.001) and 0 ppb PBB control (significance in one-sided-independent *t-*test designated by pound symbols where # is p < 0.05; ^##^ is p < 0.01; and ^###^ is p < 0.001). Centerlines of the boxplots represent the medians and box limits indicate the 25^th^ and 75^th^ percentiles which were calculated by R software; the extent of the whisker represents 1.5 times the interquartile range from the 25^th^ and 75^th^ percentiles. Outliers are shown as data points outside of the whisker range.

In the germline, all DNA methylation marks, including imprints, are erased, and imprints are re-established by *de novo* methyltransferases to reflect parent-of-origin methylation patterns. In humans, this occurs continuously at the spermatogonial stem cell stage^30^. We first wanted to determine if methylation of imprint control regions of well-studied imprint genes was altered in the sperm of men exposed to PBBs and whether or not there was a dose-related response. While the *H19* imprint control region (ICR) is paternally silenced (~90% methylated in sperm), *IGF2*, a differentially methylated region (DMR) which is hypomethylated in sperm, and *SNRPN*-ICR (~5% methylated in sperm)^31^ are maternally silenced, while *IGF2R*, a polymorphically imprinted gene in human placental tissue, is potentially active on both chromosomes. These eighty-seven participants were grouped by circulating blood levels of PBB153, and compared to the sperm of six cohort participants identified to have no detectable levels of PBB153 (0 ppb), for cohort exposure control, as well as six sperm samples from a sperm bank, as an external control (Fig. 1A–D). PBB153 levels correspond to significant decreases in *H19*-ICR DNA methylation when compared to both an internal cohort, control, and external control (Fig. 1A). Certain PBB153 concentration ranges also correspond with a significant decrease in DNA methylation in the paternally expressed genes *SNRPN-*ICR and *IGF2-*DMR, and a bi-allelically expressed gene ICR (*IGF2R*) (Fig. 1B–D). Additionally, these changes in DNA methylation were a result of PBB153 exposure, as there was no significant correlation between age and altered DNA methylation (Supplemental Fig. 1, SF1).

### PBB153 decreases methylation at ICRs of imprinting genes in vitro

While PBB153 exposure corresponds to significant reductions in DNA methylation in imprinted regions in our cohort study, we took advantage of our *in vitro* model for human spermatogenesis to further determine the mechanism by which PBB153 decreases ICR methylation. We were then able to simulate male exposure by differentiating WA01 human embryonic stem cells in the presence of 2,2′,4,4′,5,5′-hexabromobiphenyl (PBB153), into spermatogonial stem cells, primary and secondary spermatocytes and, finally, into spermatids. We previously demonstrated that this model mimics major components of human spermatogenesis^32^ and have utilized this model to examine impacts of other environmental exposures^33–35^. Additionally, our *in vitro* spermatogenesis model has been independently replicated, highlighting its reproducibility^36^. To ensure reproducibility of our current study, the production of spermatogenic lineage cells was confirmed by staining for a spermatogonial stem cell marker *PLZF/ZBTB16*, a primary spermatocyte marker *HILI/PIWIL2*, and a secondary spermatocyte marker *HIWI/PIWIL1*. A population of approximately 5% haploid cells was confirmed using FACS (Supplemental Fig. 2, SF2). The doses selected, 500 nM, 1 μM, and 5 μM correspond to 313.8 ppb, 627.6 ppb, and 3138.1 ppb, respectively. While these doses are higher than what was detected to be circulating in our human cohort at the time of our analyses, these values were chosen to more closely simulate what men would have been exposed to during the initial exposure when quarantined farm residents had a maximum measured serum PBB level of 1900 ppb^14^ and cows had levels in milk fat averaging as high as 306 ppm^37^. Higher doses were also used to better simulate a lifetime of exposure in our *in vitro* spermatogenesis model.

**Figure 2:**
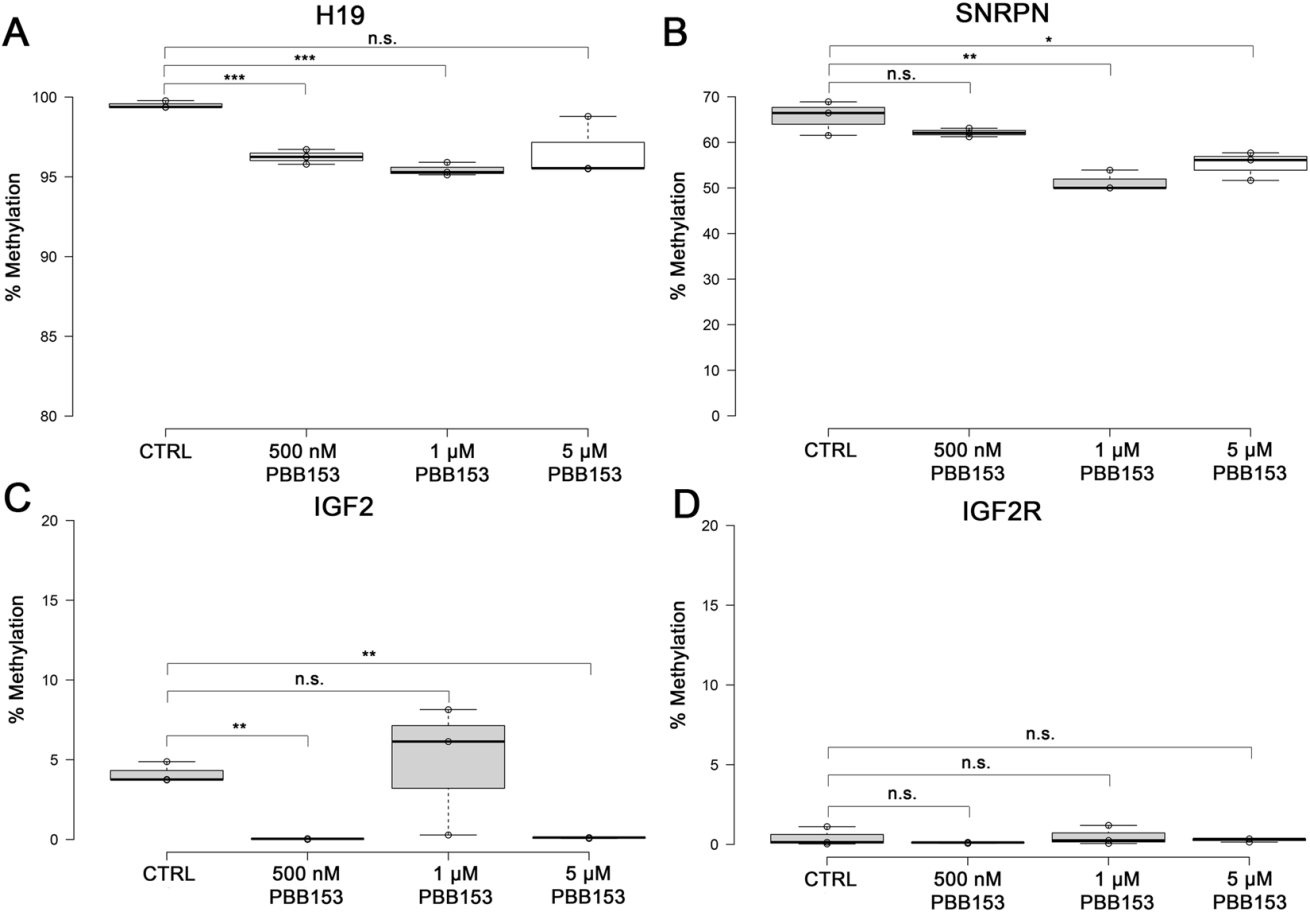
PBB153 exposure decreases methylation at the ICR of a paternally silenced gene in an *in vitro* spermatogenesis model. (**A**) Percent methylation of *H19* (paternally silenced), **(B)**. *SNRPN* (maternally silenced), **(C)**. *IGF2* (differentially methylated region, hypomethylated in sperm), and **(D)**. *IGF2R* (biallelically expressed) ICRs in response to increasing doses of PBB153. PBB153 decreases percent ICR methylation at the paternally-silenced H19 locus compared to a DMSO vehicle control. Centerlines of the box plots represent the medians and box limits indicate the 25^th^ and 75^th^ percentiles which was calculated by R software; the extent of the whiskers represent 1.5 times the interquartile range from the 25^th^ and 75^th^ percentiles. Outliers are shown as data points outside of the whisker range. N = 3 sample points for all four genes. Samples compared to determine significance using one-tailed-independent *t-*tests. Significance designated with asterisks where n.s. is not significant (p > 0.05), *is p < 0.05, **is p < 0.01, and ***is p < 0.001.

During the differentiation process, DNA methylation patterns are correctly established on at least two loci as they are *in vivo*^32^*. SNRPN* methylation remains high in this cell line after differentiation due to higher baseline methylation in undifferentiated cells compared to what is seen in the general population^31,36^. Likewise, this differentiation process mimics human exposure because, in humans, spermatogonial stem cells are located outside of the blood-testes barrier and therefore are exposed to chemicals present in the blood^38^. Using three different concentrations of PBB153 plus a vehicle control, we show that PBB153 exposure significantly reduces DNA methylation at ICRs and DMRs (Fig. 2A–D) as similarly observed in human sperm samples from the Michigan cohort. For *H19*-ICR, we see a significant decrease in methylation at both the 500 nM and 1 μM PBB153 concentrations (Fig. 2A) while at *IGF2*-DMR, we see a significant decrease in methylation at both the 500 nM and 5 μM PBB153 concentrations (Fig. 2C). While the *SNRPN*-ICR methylation level is much higher for the WA01 embryonic stem cell line compared to *in vivo* sperm, we see a significant decrease in methylation at both the 1 μM and 5 μM concentrations (Fig. 2B). Lastly, we see no change in methylation at the non-imprinting gene *IGF2R*-ICR, likely due to its already low levels of methylation (Fig. 2D).

**Figure 3:**
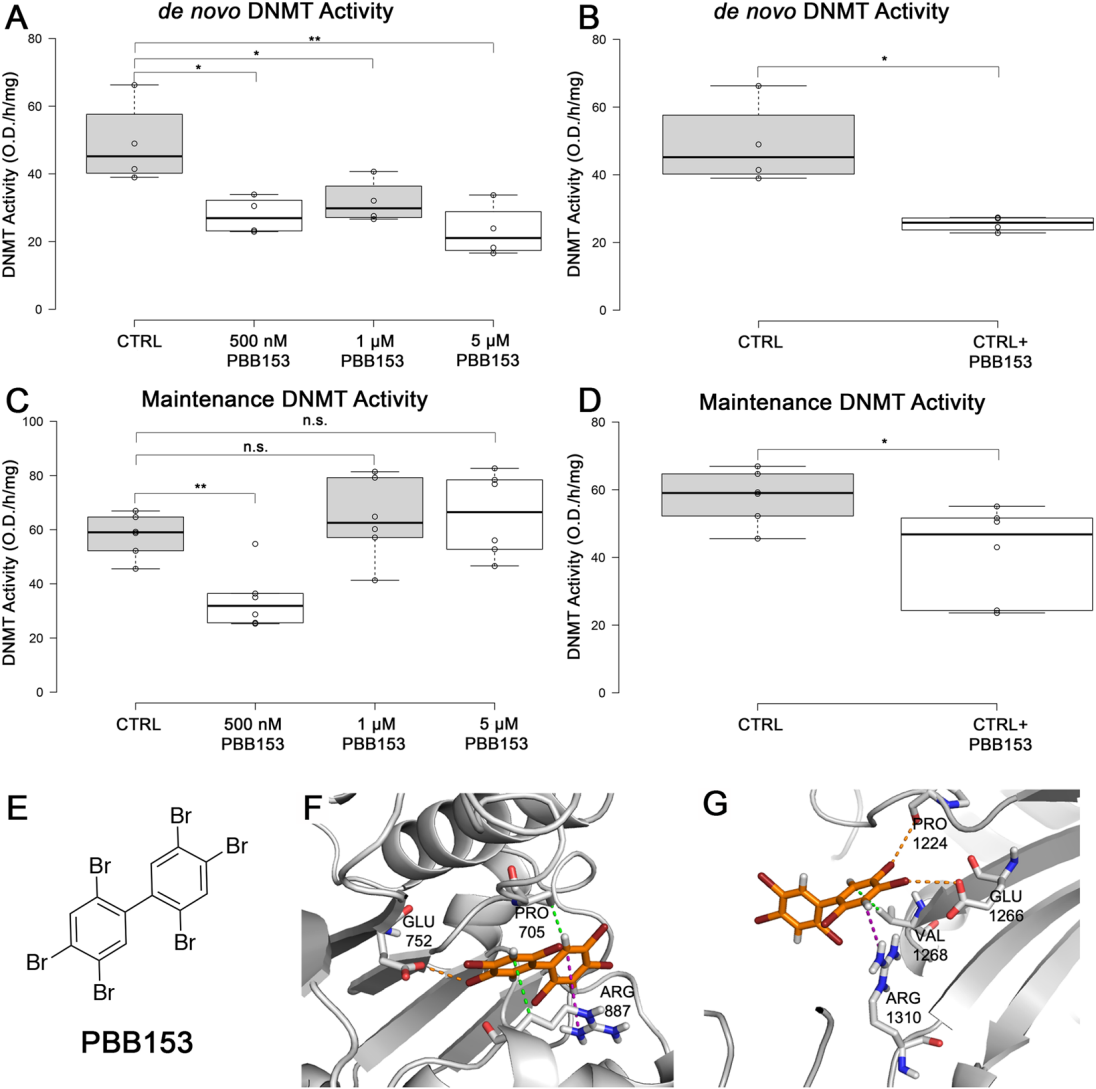
PBB153 decreases DNA methyltransferase activity during spermatogenesis and binds to the active site of DNMT3A. (**A**). *De novo* DNMT activity was measured in response to three PBB153 doses during *in vitro* differentiation of hESCs. *De novo* DNMT activity decreased significantly for all three PBB153 doses. (**B)**. *De novo* DNMT activity of DMSO control was measured in response to direct PBB153 treatment (10 µM) and significantly decreased. (**C)**. Maintenance DNMT activity was measured in response to three PBB153 doses during the *in vitro* differentiation of hESCs. Maintenance DNMT activity significantly decreased in response to the 500 nM PBB153 dose. (**D)**. Maintenance DNMT activity of DMSO control was measured in response to direct PBB153 treatment and significantly decreased. For each, significance determined by a one-tailed-independent *t*-test and designated by asterisks where *is p < 0.05; **is p < 0.01; ***is p < 0.001 and n.s. is no significance compared to DMSO control (p > 0.05). In the box plots shown in (**A**–**D**), centerlines represent the medians and box limits indicate the 25^th^ and 75^th^ percentiles which were calculated by R software; the extent of the whiskers represent 1.5 times the interquartile range from the 25^th^ and 75^th^ percentiles. Outliers are shown as data points outside of the whisker range. (**E)**. Structure of PBB153. (**F)**. Docking of PBB153 in the active site of DNA methyltransferase 3 A (PDB: 2qrv) using Achilles Blind Docking Server. PBB153 potentially interacts with amino acids in the active site and inhibits DNMT 3 A/3B from catalyzing DNA methylation reactions. The purple dashed line indicates a pi-cation interaction. The green dashed lines indicate hydrophobic interactions. The orange dashed line indicates a halogen bond. (**G)**. Docking of PBB153 in the active site of DNA methyltransferase 1 (PDB: 4wxx) using Achilles Blind Docking Server. PBB153 potentially interacts with amino acids in the active site and inhibits DNMT 1 from catalyzing DNA methylation reactions. The purple dashed line indicates a pi-cation interaction. The green dashed line indicates a hydrophobic interaction. Orange dashed lines indicate halogen bonds.

Using our *in vitro* model of human spermatogenesis, we next sought to determine whether PBB153 had an effect on either *de novo* or maintenance DNA methyltransferase activity, hypothesizing that a decrease in activity of either enzyme could explain the decreases in methylation levels observed on promoter and heterochromatic regions in peripheral blood cells^19^ and in ICRs and DMRs in our current study. Using concentrations of PBB153 from our *in vitro* study (Fig. 2), we examined whether PBB153 disrupted total DNA methyltransferase (DNMT) activity in our differentiations. After ten days of treatment, PBB153 significantly reduced the *de novo* DNMT activity in spermatogenic cells at all concentrations tested (Fig. 3A), while only the 500 nM dose significantly inhibited maintenance DNMT activity (Fig. 3C). To further verify that PBB153 directly inhibits DNMT activity, we added PBB153 (at 10 µM) directly to nuclear extracts of control samples. Direct addition of PBB153 to the DNMT activity assays significantly reduced the activity of both *de novo* and maintenance DNMT activity, although the effect was greater for *de novo* DNMT activity (Fig. 3B,D). This immediate effect of PBB153 to reduce DNMT activity suggests that PBB153 is a DNMT inhibitor with potentially more potent effects on *de novo* DNMT enzymes. Additionally, this impact on *de novo* DNA methylation appears to be specific for the germline as hESCs treated with PBB153 show no significant alterations in *H19*-ICR or *SNRPN*-ICR DNA methylation (Supplemental Fig. 3, SF3).

To further demonstrate that PBB153 directly impacts *de novo* DNMT activity to reduce DNA methylation, we examined the chemical structure of PBB153 and modeled its ability to bind in the DNMT3A, one of the *de novo* DNMT enzymes along with DNMT3B, substrate binding pocket by using a docking server. The interaction between DNMT3A and PBB153 with the highest binding free energy of −6.60 kcal/mol occurs when PBB153 binds in the enzymatic binding pocket. PBB153 binds with the catalytic residue Glu-752 via a halogen bond and additional residues in the pocket, including Pro-705 via a hydrophobic interaction and Arg-887 via pi-cation and hydrophobic interactions (Fig. 3F). When we docked PBB153 with DNMT1, we found that PBB153 interacts with the enzyme binding pocket at a lower binding free energy of –5.40 kcal/mol. PBB153 interacts with the catalytic residues Arg-1310 via a pi-cation interaction and Glu-1266 via a halogen bond interaction (Fig. 3G). PBB153 also interacts with Pro-1224 via a halogen bond interaction and Val-1268 via a hydrophobic interaction. To examine whether PBB153 could be also affecting DNA methylation levels by increasing 5mC removal, we next determined if PBB153 binds to the TET1 enzyme, a protein that catalyzes the oxidation of 5-methylcytosine so that methylation can be removed. We modeled PBB153 with the available CXXC domain of TET1^39^. PBB153 potentially binds to three areas of the CXXC domain but does not interfere with His-616, the amino acid that binds DNA, in any of the models (Supplemental Fig. 4, SF4). In all three models, the closest distance between PBB153 and His-616 is more than 4 Å, meaning that not even weak electrostatic interactions are occurring between the two molecules. PBB153 is similar in structure to a bromo-substituted derivative of a potential TET-family inhibitor known as Bobcat212. This derivative does not decrease or increase human TET-family activity^40^. Further, Bobcat212 is not capable of significantly reducing or increasing 5hmC levels in hippocampal neurons. These data suggest that PBB153 is likely not activating or inhibiting the TET family enzymes. Instead, these modeling data not only support the notion that PBB153 acts as a DNMT inhibitor but also further suggests that PBB153 is a more potent inhibitor of *de novo* DNMTs compared to maintenance DNMTs.

### PBB153 exposure alters expression of developmentally critical genes in in vitro spermatogenic cells

Symptoms experienced by those in the PBB cohort include several digestive cancers^16^ and increased odds of thyroid disease^18,20^ in addition to reproductive issues in the second generation^22–24^. Epigenetic dysregulation has been linked to cancer^41^ and is known to disrupt embryonic and placental development, translating into developmental problems later in life. DNA methylation is an epigenetic mechanism that can directly alter gene expression, and improper establishment results in several genomic imprinting disorders that cause developmental problems. It has also become widely accepted that DNA methylation patterns are sensitive to environmental exposures. PBB exposure may explain the increased risk of thyroid disease by altering DNA methylation in peripheral blood near sites of estrogen receptor transcription factors^19^. Further, altered DNA methylation in gametes, as seen in the current study, can lead to abnormal development and health problems in subsequent generations if it impacts gene expression.

**Figure 4:**
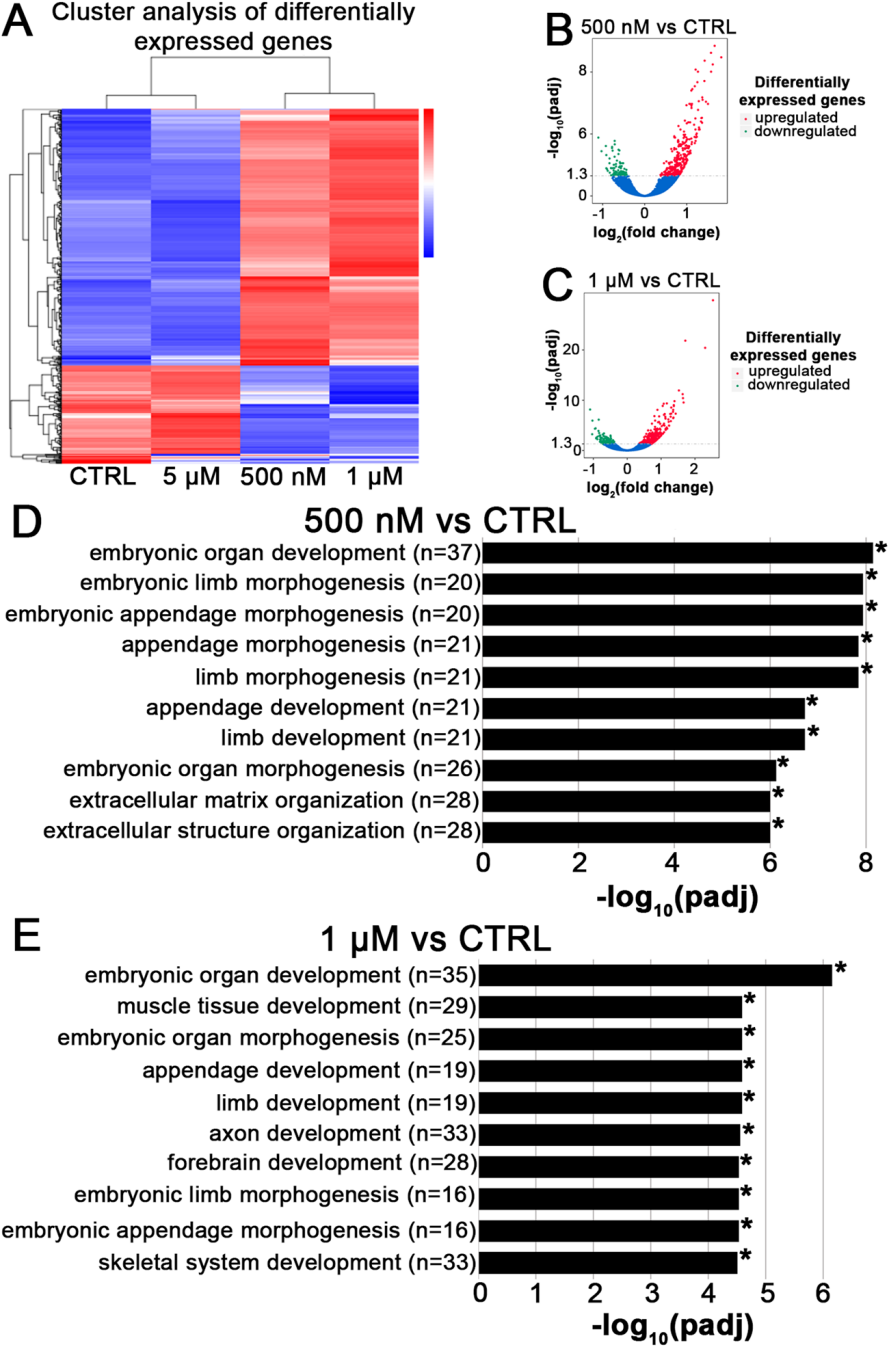
The hierarchical clustering and biological processes analysis of differentially expressed genes in *in vitro* derived spermatogenesis products exposed to PBB153. (**A**). Hierarchical clustering of differentially expressed genes in spermatids exposed to 500 nM, 1 μM and 5 μM PBB153 compared to DMSO vehicle control. (**B,C)** Volcano plots depicting −log_10_ range of differentially expressed genes, 437 genes in the 500 nM exposure sample, 342 upregulated and 95 downregulated (**B**) and 459 genes in the 1 μM exposure sample, 344 upregulated and 115 downregulated (**C**). (**D,E)**. Gene ontology (GO) analysis of the top 10 significantly enriched GO terms where * is padj < 0.05 (P-values adjusted using the Benjamini and Hochberg’s approach for controlling the false discovery rate) in the 500 nM exposure sample (**D**) and the 1 μM exposure sample (**E**).

Since exposure to PBB153 alters DNA methylation in our *in vitro* derived spermatogenic cells, we hypothesized that lower levels of epigenetic gene inactivation would, in turn, alter gene expression. Using our *in vitro* model for human spermatogenesis with a feeder-free modification previously reported^36^, we generated spermatogenic cells in the presence of the same three concentrations of PBB153 and a vehicle control used in the previous experiments, isolated the total RNA and performed RNAseq analyses. The doses 500 nM and 1 μM PBB153 had the most significant effects, resulting in dramatic alterations in gene expression when compared to the control and 5 μM PBB153 dose (Fig. 4A). Compared to the control, the 500 nM sample had 437 significant differentially expressed genes, 342 which were upregulated and 95 which were downregulated (Fig. 4B), the 1 μM sample had 459 significant differentially expressed genes, 344 which were upregulated and 115 which were downregulated (Fig. 4C), and the 5 μM sample had just 4 genes, all of which were significantly downregulated (Supplemental Fig. 5, SF5). Interestingly, at 5 μM PBB153, the gene expression profile in the differentiation products appears to be very similar to that of the DMSO control (Fig. 4A, SF5), highlighting a concentration-dependent impact on gene expression.

Further gene ontology (GO) analyses reveal common themes among the differentially expressed genes for both 500 nM and 1 μM PBB153 compared to the control. The genes identified to have significant differential expression are essential for proper embryo development including genes involved in the proteinaceous extracellular matrix, extracellular structure organization, cell-cell junctions, mesoderm development, embryonic organ development, limb morphogenesis and development, appendage morphogenesis and development, embryonic organ morphogenesis, and mesoderm development (Fig. 4D,E, additional significantly enriched GO analyses are shown in Supplemental Tables, ST 1–3). At the 500 nM dose, genes responsible for cotranslational protein targeting, protein targeting to the endoplasmic reticulum, cytosolic ribosome, cell-cell junction, and respiratory system development were significantly altered (Fig. 4D, ST1). At the 1 μM dose, genes responsible for embryonic axon development, forebrain development, axonogenesis, sensory organ morphogenesis, muscle tissue development, and eye development were significantly altered (Fig. 4E, ST2). While we cannot confirm that developmental defects such as the ones described by GO analyses in this study were the cause of the increased miscarriages in the daughters of women in the PBB cohort, such consistently high alterations in gene expression in developmentally critical genes could reasonably cause significant growth alterations leading to non-viable pregnancies.

## Discussion

Over the course of what has since been referred to as “Cattlegate,”^8^ approximately 6.5 million people in the state of Michigan were exposed to PBBs from animal products. Though it has been nearly 45 years since the accident, people who were exposed through the consumption of contaminated animal products and those exposed *in utero* and through breastmilk still have circulating levels of PBBs. As recently as 2015, 60% of tested members of the PBB Registry have elevated levels of PBB above the ninety-fifth percentile of the U.S. population^42^. The half-life of PBB is estimated to be between 10 and 29 years^24,43^, which may explain why people are still experiencing health issues related to this accident. Additionally, the structure of PBBs is similar to other, more widespread endocrine-disrupting chemicals (EDCs) such as PCBs and PBDEs, on which, little is known about the effects of exposure on the germline. Thus continuing to understand the long-term ramifications of PBB153 exposure is of the utmost importance for those in Michigan, and may also shed light on the potential intergenerational impacts of exposure to other EDCs. With its ability to impact the sperm epigenome, our work demonstrates how PBB153 exposure may have long-lasting effects, not only on people directly exposed but their children, as well.

In this study, we found that circulating levels of PBB153 is associated with decreased methylation at ICRs of imprinted genes in the sperm of men in the Michigan PBB Registry. Using our novel *in vitro* human spermatogenesis model, we found that PBB153, the primary PBB compound involved in the massive exposure, mimics the *in vivo* phenotype after just 10 days of exposure and that this is likely due to PBB153 binding to catalytic binding pocket of *de novo* methyltransferases to lower the activity of the DNMT enzymes, although PBB153 also can affect maintenance methyltransferase activity. This phenomenon has the potential to cause diseases in offspring. For example, a reduction in DNA methylation in sperm may lead to the overexpression of imprinted genes that should be silenced and translate to improper parent-of-origin gene imprinting in offspring. This hypomethylation can lead to gene imprinting diseases such as Silver-Russell Syndrome. Imprinted genes are essential for fetal growth^44^ and play an important role in other aspects of development^45^. Modified inheritance of critical imprinted genes could explain the mismatch in symptoms experienced in the two generations of PBB153 affected individuals. Additionally, the results from this study show that the dose of PBB153 plays a critical role in the alteration of gene expression in spermatogenic cells. Our data show that the lower doses tested (500 nM and 1 µM) induce the most significant changes in gene expression compared to the highest dose tested (5 µM) which shows a similar gene expression profile to control samples. This specific, but complex relationship between dose and effect may explain the difficulty in finding consistent results in human epidemiological studies as well as the variety of different health outcomes reported within the PBB cohort.

The effect of improper establishment of genetic imprints during spermatogenesis is illustrated in the altered gene expression for genes essential for proper embryonic development. This may explain why the children of people directly exposed to PBB during the initial exposure have different health effects compared to their parents, including reproductive problems. However, it is possible that the endocrine-disrupting nature of PBB153 may also play a role in the alteration of gene expression seen in our study. Additionally, we could not simulate a lifetime of exposure that many of these cohort members have experienced, but by exposing human embryonic stem cells to PBB153 as they differentiate into spermatogenic cells at doses slightly higher than what persists in the population for ten days, we aim to model the *in vivo* effects of decades of exposure which is expected to act through the same mechanism.

The interplay between epidemiology, epigenetics, and *in vitro* models is an important one for improving the study of human health and associated risks. Epigenetics can explain trends in epidemiological studies^46^, but by including *in vitro* modeling, we can better determine the underlying mechanisms affecting human health. With information from epidemiological studies, the use of *in vitro* experiments allows us to more accurately model exposures and elucidate how epigenetic modifications play a role in gene-environment interactions. This model for human spermatogenesis aims to take modeling exposures a step further by examining paternal pre-conception exposures with the hopes of learning more about how the exposome of the father may impact offspring health. We hope this study can establish a framework for encouraging the integration of future epidemiology work with epigenetic studies and relevant human *in vitro* studies in order to obtain a more holistic view of human disease, primarily as it corresponds to exposures preceding conception and birth. Multi-faceted approaches, such as the one conducted in this study, should serve as a model platform for uncovering novel environmental contributions to Developmental Origins of Health and Disease.

## Methods

### Human samples and DNA isolation

This study examined participants who had previously enrolled in the Michigan PBB Registry (IRB# 691-2002). Participants were presented with the study’s details by staff from Emory University Rollins School of Public Health who also received the men’s informed consent upon agreement to partake in the study. Each participant was assigned a unique study identification number.

Staff from Emory University gave each participant a semen collection kit consisting of a semen collection jar, instructions, and a thermos to insulate the collection jar. The men in the study were asked to abstain from ejaculation for 2 days prior to semen collection but were allowed to participate if they had not abstained for 2 days. A sample of blood was collected from each participant via venipuncture by a trained phlebotomist which was used for measurement of PBB levels.

Analyses for PBB levels of the participants included assaying the serum samples for congener-specific PBBs, PCBs, and PBDEs and lipids using gas chromatography-tandem mass spectrometry which targeted brominated compounds as described in Marder, *et al*.^47^. Some participants enrolled in the Michigan PBB Registry were not found to have PBB circulating as determined by serum analysis. The semen samples from these participants were included as controls.

Semen samples used as unexposed controls were obtained from Xytex Cryo International. Samples from Xytex came from donors with no ties to Michigan to sampling donors unknowingly affected by PBB exposure. Samples were stored and thawed according to company’s instructions. DNA was collected from sperm and *in vitro* spermatids using the DNeasy Blood and Tissue Kit according to manufacturer’s instructions (QIAGEN, Germantown, MD). Sperm samples were washed twice with 70% ethanol prior to DNA isolation, and 7.8ul of 2 M dithiothreitol was added during lysis step. DNA concentration was determined using a Qubit 3.0 with a Qubit dsDNA HS Assay Kit (Thermo Fisher Scientific) or DeNovix dsDNA High Sensitivity Assay Kit (DeNovix, Inc.) and an Epoch™Δ Microplate Spectrophotometer with a Take3 plate (Epoch Life Science). For quality control, DNA samples were only used in further analyses if the ratio of the absorbance at 260 nm to absorbance at 280 nm ratio was greater than 1.7.

### Cell culture

NIH-approved WA01 (H1, WiCell) male, human embryonic stem cells were maintained as described^32,34^. hESCs were pasaged using ReLeSR (STEMCELL Technologies) and plated onto Matrigel (Corning Life Sciences) coated plates as described^32,34^. Differentiation into spermatogenic lineages were conducted as previously described^32–35^ with mouse STO cells for feeder cells or directly onto Matrigel. Medium was gassed every other day with a blood gas mixture (5% carbon dioxide, 5% oxygen, and 90% nitrogen). PBB153 (2,2′,4,4′,5,5′-Hexabromobiphenyl, AccuStandard) was dissolved in DMSO and was diluted to final concentrations of 500 nM, 1 μM, and 5 μM in spermatogonial stem cell (SSC) medium. PBB153 or DMSO (vehicle control) was continuously added to SSC media beginning on day 1 of the differentiation.

Zhao, *et al*.^36^ showed that pluripotent stem cells could be differentiated into spermatogonia-like cells without the use of STO feeder cells. The germ cell differentiations conducted in this study were directly on Matrigel with a minority of differentiations on STO feeder cells. All differentiations were carried out using the SSC medium from Easley IV, *et al*.^32^, with the following modification: replacing putrescine and beta-mercaptoethanol with ascorbic acid as described^36^.

### Cell Sorting

On Day 10 of the differentiation protocol, cells were collected using TrypLE™Δ Express (ThermoFisher) and were prepared in order to isolate haploid cells during fluorescence-activated cell sorting (FACS). The nuclei of live cells were stained using RedDot™Δ1 Far-Red Nuclear Stain (Biotium, Inc.) according to manufacturer’s instructions in SSC medium in order to observe the presence of a haploid peak. Prepared samples were run on a FACS Aria II or Diva sorter (BD Biosciences). DNA was then isolated from collected haploid cells for DNA methylation analysis.

### DNA methylation assessment

Following genomic DNA isolation from semen samples and collected haploid cells grown *in vitro*, the methylation status was assessed at the imprint control regions *H19, IGF2, IGF2R*, and *SNRPN*. Isolated DNA was used to prepare restriction digests using the Epitect Methyl II DNA Restriction kit according to manufacturer instructions (QIAGEN). Digested DNA was amplified using the QIAGEN EpiTect Methyl II PCR Array (QIAGEN) with the following primers: *H19* (EPHS102101-1A), *IGF2* (EPHS102104-1B), *IGF2R* (EPHS112868-1A) and *SNRPN* (EPHS104389-1A). Methylation at each imprint control region was determined by inputting each sample’s raw C_T_ values into the “Raw Data” tab of the “EpiTect Methyl II 96-Well Complete Data Analysis Spreadsheet” provided by the manufacturer. For sperm methylation data, Xytex, n = 6; 0 ppb PBB, n = 6; 0.005–0.03 ppb PBB, n = 21; 0.031–0.09 ppb PBB, n = 21; 0.104–0.275 ppb PBB, n = 17; 0.287–0.597 ppb PBB, n = 15; 1.034–50.369 ppb PBB, n = 7. Each n represents a single PCR analysis per gene for each DNA sample taken from a sperm sample from each PBB Registry donor and each Xytex Cryo International donor. For in vitro derived spermatogenic cells, each n represents a biological replicate; haploid DNA from three separate differentiations was used to run a PCR array for each gene. For hESC analysis in **SF4**, N = 4 sample points for each gene that represent two digests of the same DNA sample isolated from hESCs treated with each dose of PBB153 and a vehicle control with three PCR arrays of one set of digests and one PCR array of the other digested DNA.

### mRNA sequencing

Differentiations were carried out as previously described to yield three biological replicates. RNA was extracted from differentiations using RNEasy RNA isolation kit per manufacturer’s instructions (QIAGEN). RNA samples were then sent to Novogene (Novogene Corporation, Inc.) for RNA-seq analyses.

Similar analyses using Novogene’s services are shown^48–50^. RNA-seq data (Fig. 4, Supplemental Fig. 3, and Table 1) that support the findings of this study have been deposited in the NCBI SRA BioProject database, ID: PRJNA628711.

#### Data analysis

Novogene checks RNA quantity and quality in three ways: Nanodrop (OD260/OD280), agarose gel electrophoresis to confirm RNA integrity and to check for potential contamination, and Agilent 2100 as a second check of RNA integrity. All samples sent off for sequencing passed Novogene’s quality control. The work flow proceeded as follows: mRNA was isolated from total RNA using poly-T oligo-attached magnetic beads. mRNA was then fragmented using fragmentation buffer. The first cDNA strand was synthesized using random hexamer primers and M-MuLV reverse transcriptase. The second strand was then generated using DNA Polymerase I and RNase H. The double stranded cDNA was purified using AMPure XP beads. Any remaining overhangs present in the ds-cDNA were converted to blunt ends using exonuclease and polymerase enzymes. The 3′ ends of DNA fragments were adenylated and then ligated to the NEBNext Ultra RNALibrary Prep Kit Adaptors (RNA 5′ adaptor: 5′ AATGATACGGCGACCACCGAGATCTACACTCTTTCCCTACACGACGCTCTTCCGATCT; RNA 3′ adaptor: 5′ GATCGGAAGAGCACACGTCTGAACTCCAGTCACATCACGATCTCGTATGCCGTCTTCTGCTTG) with PCR primers complementary to sequences on the flow cell used for sequencing in order to prepare for hybridization. The fragments were then purified using the AMPure XP system (Beckman Coulter). The library was then generated via PCR amplification and the products purified using AMPure XP beads. The library was assessed quantitatively using a Qubit 2.0 Fluorometer, Agilent 2100 (Agilent) in order to detect and confirm the insert size of 250–300 bp, and finally the effective concentration of the library was determined using qPCR. The libraries were pooled and sequenced using an Illumina NovaSeq or HiSeq with long read length paired-end 150 bp (PE150) sequencing. From the Illumina instrument, the raw data files were transformed into sequenced reads by CASAVA base recognition (base calling). Low quality adaptors and reads with adaptors were filtered out in order to leave only clean reads. STAR (v2.5) software was used to align paired-end clean reads to the reference genome (*Homo sapiens* hg19, downloaded from genome website browser NCBI/UCSC/Ensembl) using the method of Maximal Mappable Prefix (MMP) to generate precise mapping results for junction reads.

#### Quantification of gene expression level

Gene expression was estimated by the abundance of transcripts (sequenced read counts) mapped to the genome using HTSeq (v0.6.1). Fragments Per Kilobase of transcript sequence per Million base pairs sequenced (FPKM) was used to estimate gene expression levels, taking into account sequencing depth and gene length of fragment counts as it is the most commonly used method for estimating gene expression levels^51^.

#### Differential expression analysis

For each group of samples (three distinct biological replicates) treated with PBB153, differential expression analysis comparing to the control samples (three biological replicates) was performed using the DESeq. 2 R package (2_1.6.3) which determines statistical differential expression using a negative binomial distribution model. The resulting p values were adjusted using the Benjamini and Hochberg approach for controlling false discovery rate. Genes with an adjusted p value < 0.05 as determined by DESeq. 2 were determined to be differentially expressed.

### Nuclear Extraction and DNMT activity assays

To assess activity of DNMT enzymes during *in vitro* spermatogenesis with exposure to different doses of PBB153, nuclear extracts were isolated using Nuclear Extraction kit per manufacturer’s instructions (Active Motif). To examine the activity of the DNMT enzymes, the isolated nuclear extracts were used in the DNMT Activity/Inhibition Assay per manufacturer’s instructions (Active Motif). The activity of the *de novo* DNMT enzymes was obtained by incubating the nuclear extracts in Complete Enzymatic Buffer AM1 (Active Motif) for 2 hours while the activity of the maintenance DNMT enzymes was obtained by incubating for 1 hour in accordance with manufacturer’s instructions. To directly examine the effect of PBB153 on the DNMT enzymes, PBB153, at a final concentration of 10 μM, was added to control nuclear extract prior to the initial incubation step. DNMT activity was determined by following the equation provided by the manufacturer (Active Motif). N = 4 in Fig. 3A,B, where each n represents an average of 6 measurements from the nuclear extract obtained from individual differentiations and n = 6 sample points in Fig. 3C,D where each n represents six replicates obtained from one differentiation.

### Computational Modeling

To determine how PBB153 interacts with DNMT and TET enzymes, the chemical structure of PBB153 was entered into the Achilles Blind Docking Server (available at: http://bio-hpc.eu/software/blind-docking-server/) as a ligand for the receptor DNMT 3 A/3 L (PDB: 2qrv), DNMT1 (PDB: 4wxx), and TET1 (PDB: 6asd). Docking calculations were performed across the entire protein surface to determine regions where binding is energetically favorable. The Achilles Blind Docking Server uses a custom version Autodock Vina (Vina_vision) to run the calculations^52^.

### Immunocytochemistry

Cells cultured in conditions previously described were fixed in 4% paraformaldehyde (Electron Microscopy Sciences) for fifteen minutes and then blocked with buffer containing 1X phosphate-buffered saline solution (PBS) (Fisher Scientific), 0.1% Triton X (Sigma), 5% bovine serum albumin (BSA) (Fisher Scientific) overnight at 4 °C. Primary antibody incubation occurred overnight at 4 °C in blocking buffer followed by three washes in 1X PBS with 0.1% Triton-X for ten minutes each at room temperature. Secondary antibody (Invitrogen, 1:2000 dilution) incubation was performed at 37 °C for two hours followed by three washes as described above. Samples were co-stained with Vectashield mounting medium with DAPI (Vector Laboratories, Inc.). Antibodies used were PLZF/ZBTB16 (R&D System, MAB2944), HILI/PIWIL2 (Abcam, AB181340) and HIWI/PIWIL1 (Abcam, AB105393).

## Supplementary information

Supplementary Information.
