# Supplementary material for: Detrimental effects of flame retardant, PBB153, exposure on sperm and future generations
Source: Sci Rep. 2020 May 22;10:8567. doi: 10.1038/s41598-020-65593-x (PMC7244482; doi:10.1038/s41598-020-65593-x)
Supplement: Supplementary file 1 — Supplementary Information. [file 41598_2020_65593_MOESM1_ESM.docx]

**Supplemental Figures and Tables**

**Detrimental effects of flame retardant, PBB153, exposure on sperm and future generations**

Katherine Watkins Greeson*^1,2^, Kristen L. Fowler*^1,2^, Paige M. Estave^1,2^, S. Kate Thompson^1,2^, Chelsea Wagner^3^, R. Clayton Edenfield^1,2^, Krista M. Symosko^1,2^, Alyse N. Steves^2,4^, Elizabeth M. Marder^5^, Metrecia L. Terrell^5^, Hillary Barton^5^, Michael Koval^6^, Michele Marcus^5^, Charles A. Easley IV^1,2,4#^

^*Signifies that authors contributed equally^

^#Signifies corresponding author^

^1^Department of Environmental Health Science, College of Public Health, University of Georgia, Athens, GA

^2^Regenerative Bioscience Center, University of Georgia, Athens, GA

^3^Department of Obstetrics, Gynecology and Reproductive Sciences, McGovern Medical School at The University of Texas Health Science Center at Houston, Houston, TX

^4^Neuropharmacology and Neurologic Diseases, Yerkes National Primate Research Center, Atlanta, GA

^5^Department of Epidemiology, Rollins School of Public Health, Emory University, Atlanta, GA

^6^Division of Pulmonary, Allergy, Critical Care and Sleep Medicine, Department of Cell Biology, Emory University School of Medicine, Atlanta, Georgia


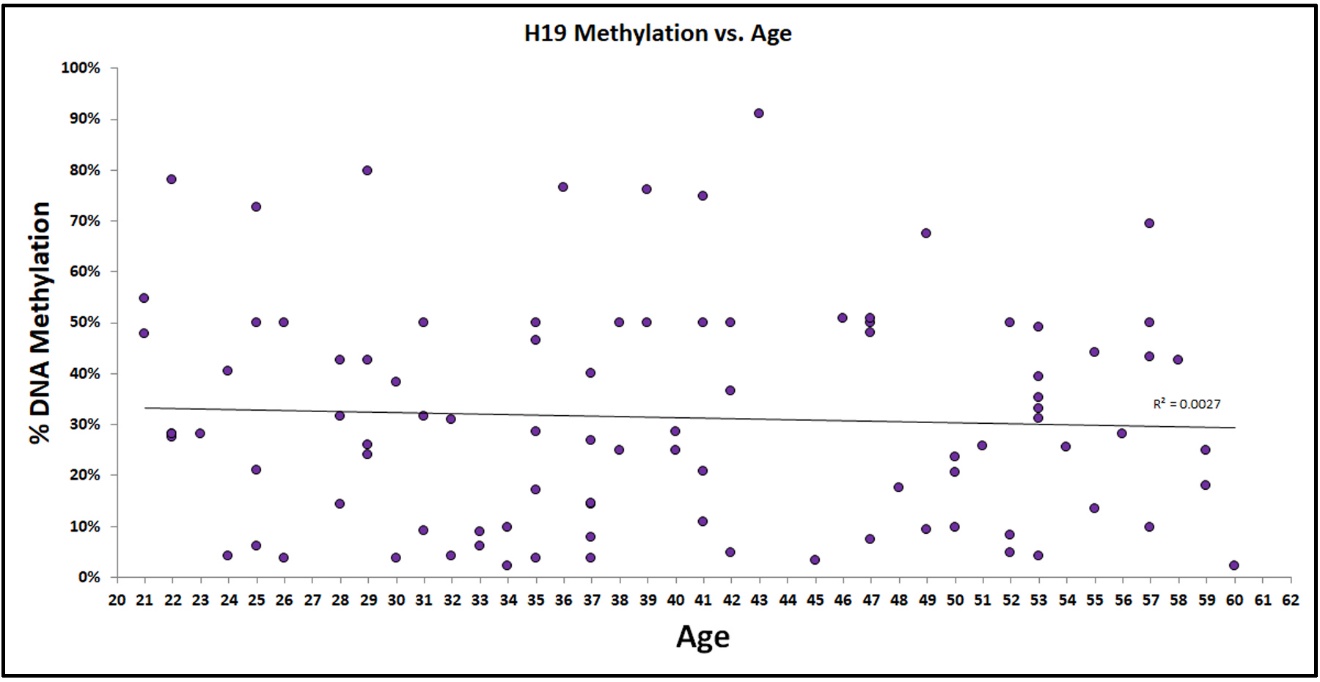


**Supplemental Figure 1. *H19* Methylation shows no correlation with age.** Individual *H19* methylation levels in 93 participants from the PBB Registry are shown. A correlation value (R^2^ between age (x-axis) and % DNA methylation (y-axis) is shown. There is no correlation between decreased *H19* methylation and age.


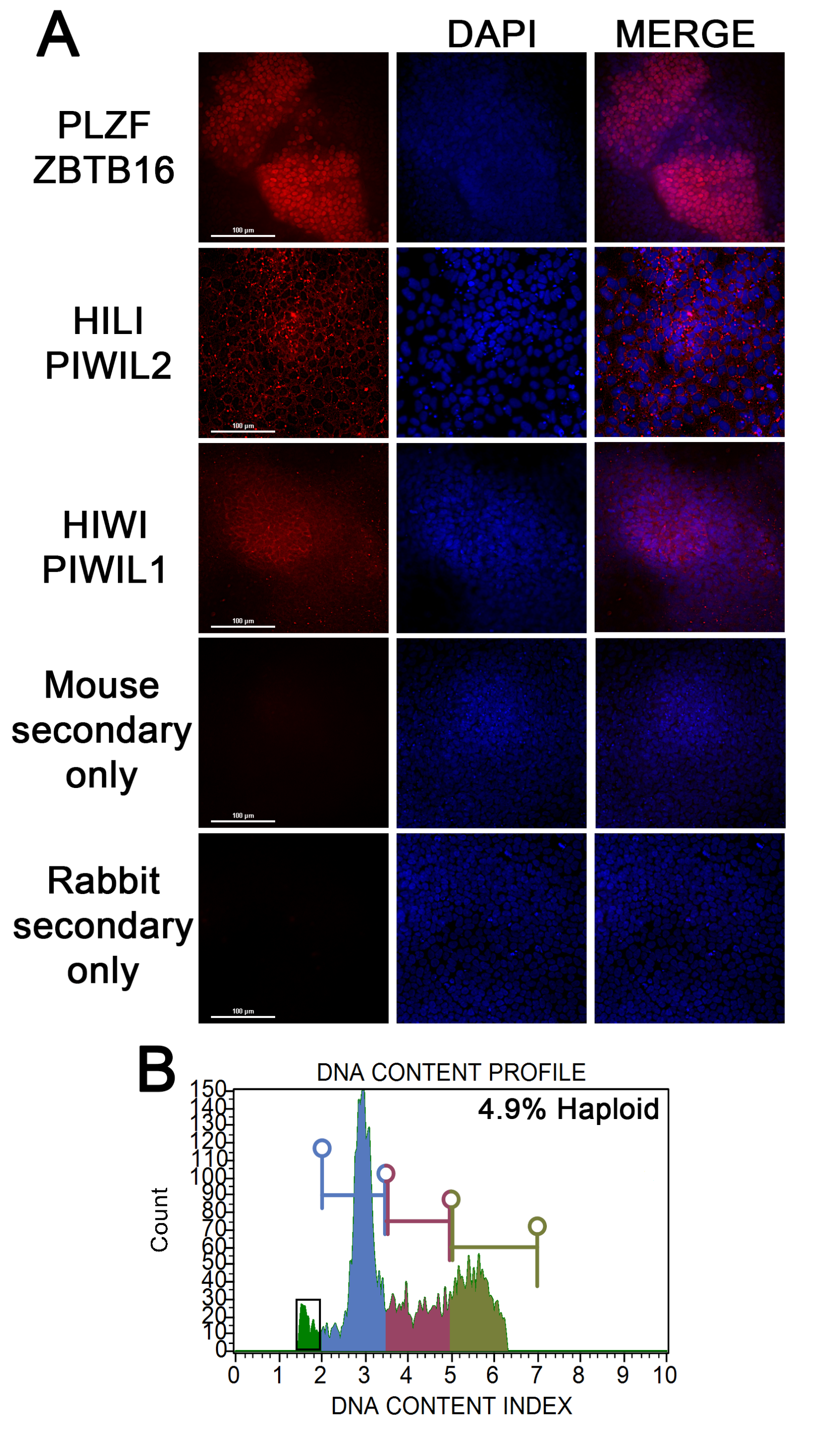


**Supplemental Figure 2. Differentiation of human pluripotent stem cells yields protein expression of germ cell-specific cell markers and a population of haploid cells. A.** H1 ESCs cultured in SSC conditions for 10 days and then stained for PLZF/ZBTB16, HILI/PIWIL2, and HIWI/PIWIL1 with secondary antibody-only controls shown. DNA labeled with DAPI. Scale: 100 μm. **B.** A representative graphical analysis of the cell cycle profile is shown with the haploid population, consisting of about 5% of the cell population, indicated with a black rectangle to show the population used for gating in FACS experiments.


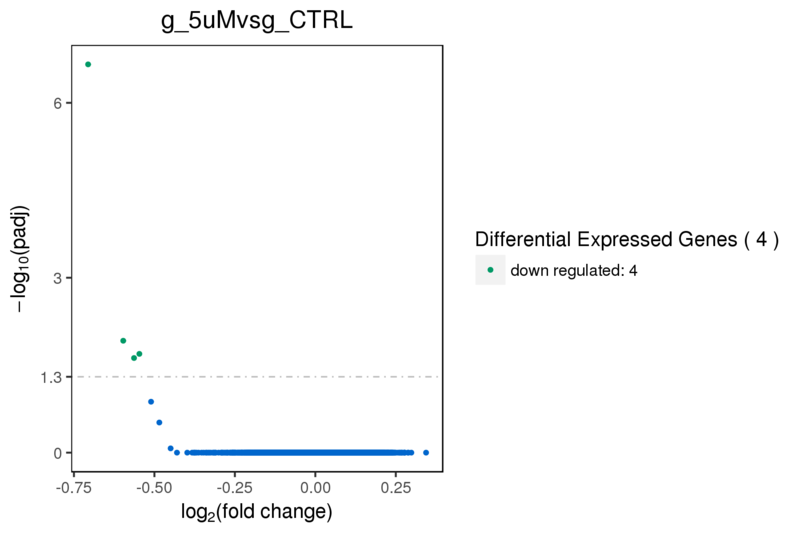


**Supplemental Figure 3. The differentially expressed genes in *in vitro* derived spermatogenesis products exposed to 5 μM PBB153.** Volcano plot depicting -log_10_ range of differentially expressed genes, 4 genes were significantly downregulated.


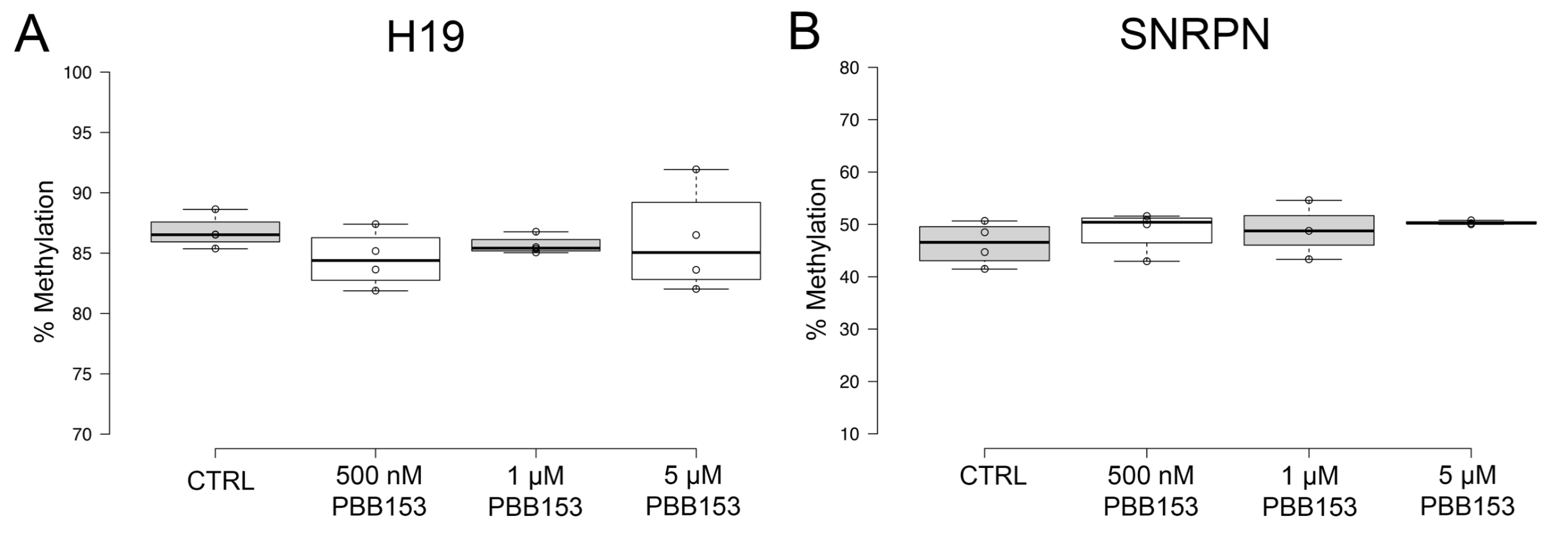


**Supplemental Figure 4. Treatment of human embryonic stem cells with PBB153 does not induce changes in DNA methylation at differentially methylated regions H19 and SNRPN. A.** Percent methylation of *H19* and **B.** *SNRPN* in H1 ESCs after maintenance in mTeSR Plus cell culture media with 500 nM, 1 μM, or 5 μM PBB153 added or DMSO as a vehicle control. After five days of treatment, representing a growth period for the ESCs, DNA was harvested, and methylation analyses conducted. Percent methylation of *H19* and *SNRPN* were unchanged compared to a DMSO vehicle control. Centerlines of the boxplots represent the medians and box limits indicate the 25^th^ and 75^th^ percentiles which was calculated by R software; the extent of the whiskers represent 1.5 times the interquartile range from the 25^th^ and 75^th^ percentiles. Samples were compared to determine significance using one-tailed-independent *t*-tests.


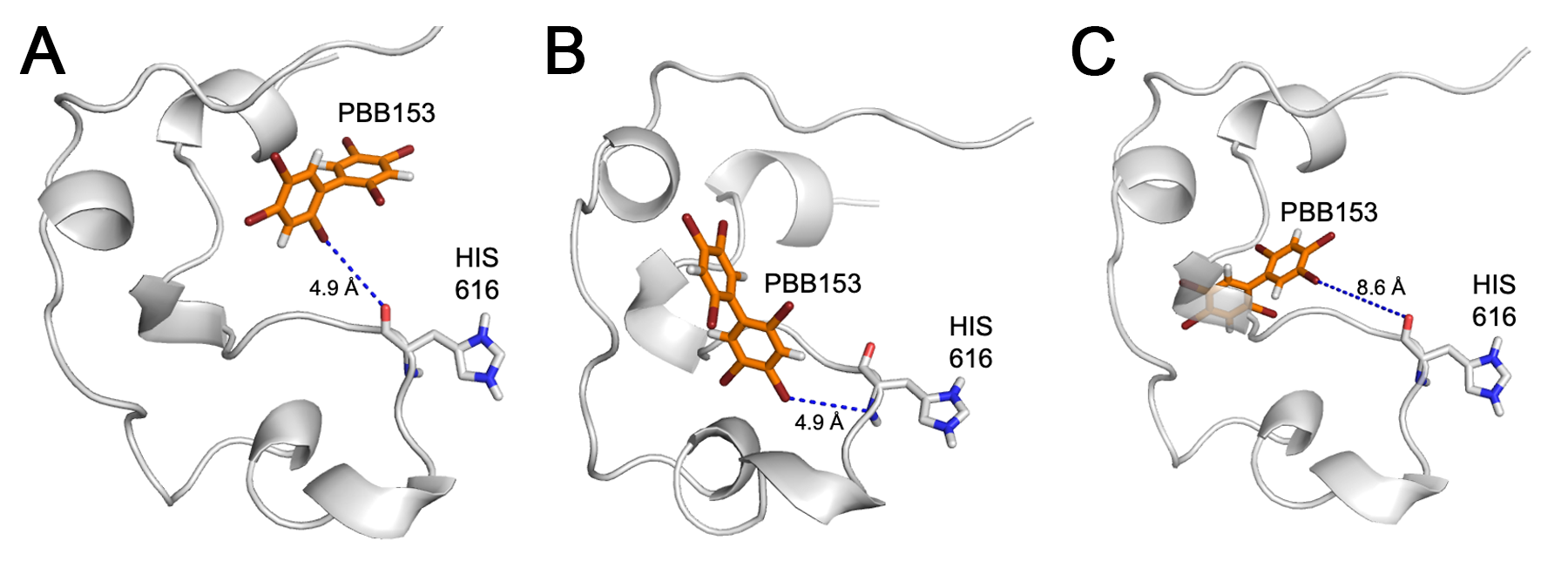


**Supplemental Figure 5. PBB153 may bind to the CXXC domain of TET1 but does not interact with the DNA-binding amino-acid.** **A.** First docking site on the TET1 CXXC binding motif (PDB: 6asd) using Achilles Blind Docking Server. PBB153 is 4.9 Å away from His-616 shown as a blue dashed line. **B.** Second docking site, showing that PBB153 is 4.9 Å away from His-616. **C.** Third docking site, showing that PBB153 is 8.6 Å away from His-616.

**Table 1. 500nM PPB153**

| Description | ID | pvalue | padj | Count | Category | geneID |
| --- | --- | --- | --- | --- | --- | --- |
| embryonic organ development | GO:0048568 | 1.99E-12 | 7.18E-09 | 37 | BP | DLX6/FOXC1/PRDM1/VASH1/ITGA8/GRHL2/EYA1/MEGF8/HPN/DLX5/  RARRES2/PRRX1/RDH10/NES/MYCN/TBX3/TFAP2A/MYO7A/STRA6/  WNT9A/KIT/WNT9B/FOXH1/PKDCC/EOMES/PITX2/CTHRC1/IGF2/C2CD3  /EFNA1/RARG/HOXB2/IRX5/ALDH1A3/SLITRK6/PBX1/PDGFA |
| embryonic limb morphogenesis | GO:0030326 | 9.56E-12 | 1.15E-08 | 20 | BP | DLX6/PITX1/TP63/GRHL2/MEGF8/DLX5/DKK1/ZBTB16/PRRX1/RDH10/  MYCN/TBX3/TFAP2A/CRABP2/WNT9A/PITX2/SP8/C2CD3/RARG/PBX1 |
| embryonic appendage morphogenesis | GO:0035113 | 9.56E-12 | 1.15E-08 | 20 | BP | DLX6/PITX1/TP63/GRHL2/MEGF8/DLX5/DKK1/ZBTB16/PRRX1/RDH10/  MYCN/TBX3/TFAP2A/CRABP2/WNT9A/PITX2/SP8/C2CD3/RARG/PBX1 |
| appendage morphogenesis | GO:0035107 | 2.01E-11 | 1.45E-08 | 21 | BP | DLX6/PITX1/TP63/GRHL2/MEGF8/DLX5/DKK1/ZBTB16/PRRX1/RDH10/  MYCN/TBX3/TFAP2A/CRABP2/WNT9A/PKDCC/PITX2/SP8/C2CD3/RARG/  PBX1 |
| limb morphogenesis | GO:0035108 | 2.01E-11 | 1.45E-08 | 21 | BP | DLX6/PITX1/TP63/GRHL2/MEGF8/DLX5/DKK1/ZBTB16/PRRX1/RDH10/  MYCN/TBX3/TFAP2A/CRABP2/WNT9A/PKDCC/PITX2/SP8/C2CD3/RARG/  PBX1 |
| appendage development | GO:0048736 | 3.71E-10 | 1.91E-07 | 21 | BP | DLX6/PITX1/TP63/GRHL2/MEGF8/DLX5/DKK1/ZBTB16/PRRX1/RDH10/  MYCN/TBX3/TFAP2A/CRABP2/WNT9A/PKDCC/PITX2/SP8/C2CD3/RARG/  PBX1 |
| limb development | GO:0060173 | 3.71E-10 | 1.91E-07 | 21 | BP | DLX6/PITX1/TP63/GRHL2/MEGF8/DLX5/DKK1/ZBTB16/PRRX1/RDH10/  MYCN/TBX3/TFAP2A/CRABP2/WNT9A/PKDCC/PITX2/SP8/C2CD3/RARG/  PBX1 |
| embryonic organ morphogenesis | GO:0048562 | 1.67E-09 | 7.50E-07 | 26 | BP | DLX6/ITGA8/GRHL2/EYA1/MEGF8/HPN/DLX5/PRRX1/RDH10/MYCN/TBX3/  TFAP2A/MYO7A/STRA6/WNT9A/WNT9B/FOXH1/PITX2/CTHRC1/C2CD3/  EFNA1/RARG/HOXB2/IRX5/ALDH1A3/SLITRK6 |
| extracellular matrix organization | GO:0030198 | 2.60E-09 | 1.00E-06 | 28 | BP | DCN/TLL1/CDH1/TNC/FOXC1/FBLN1/ITGA8/ADAMTS2/COL9A3/FERMT1/  CRISPLD2/HPN/ITGB6/NID1/SPP1/TGFBI/CTSV/COL6A2/HAPLN1/CARMIL2/  FGFR4/COL6A3/ITGA2/HTRA1/HAS2/EFEMP2/HPSE2/PDGFA |
| extracellular structure organization | GO:0043062 | 2.78E-09 | 1.00E-06 | 28 | BP | DCN/TLL1/CDH1/TNC/FOXC1/FBLN1/ITGA8/ADAMTS2/COL9A3/FERMT1/  CRISPLD2/HPN/ITGB6/NID1/SPP1/TGFBI/CTSV/COL6A2/HAPLN1/CARMIL2/  FGFR4/COL6A3/ITGA2/HTRA1/HAS2/EFEMP2/HPSE2/PDGFA |
| SRP-dependent cotranslational protein targeting to membrane | GO:0006614 | 1.55E-07 | 5.09E-05 | 13 | BP | RPL31/RPL24/RPS6/RPS11/RPL32/RPL9/RPS14/RPL27A/RPS7/RPL35A/  RPL12/RPL39/RPS18 |
| mesoderm development | GO:0007498 | 2.05E-07 | 6.14E-05 | 15 | BP | MATK/FOXC1/EYA2/TP63/ITGA8/EYA1/LHX2/DKK1/TBX3/POU4F1/FOXH1/  EOMES/ITGA2/IRX3/MIXL1 |
| skeletal system development | GO:0001501 | 2.64E-07 | 7.32E-05 | 31 | BP | DLX6/TLL1/FOXC1/PITX1/TP63/GRHL2/EYA1/MEGF8/DLX5/ZBTB16/PRRX1/  TGFBI/RDH10/FGFRL1/MYCN/TBX3/TFAP2A/WNT9A/MEIS1/PDGFC/HAPLN1/  KIT/WNT9B/PKDCC/PITX2/IGF2/HAS2/RARG/HOXB2/IRX5/PBX1 |
| cotranslational protein targeting to membrane | GO:0006613 | 2.95E-07 | 7.59E-05 | 13 | BP | RPL31/RPL24/RPS6/RPS11/RPL32/RPL9/RPS14/RPL27A/RPS7/RPL35A/RPL12/  RPL39/RPS18 |
| protein targeting to ER | GO:0045047 | 4.78E-07 | 0.000115 | 13 | BP | RPL31/RPL24/RPS6/RPS11/RPL32/RPL9/RPS14/RPL27A/RPS7/RPL35A/RPL12/  RPL39/RPS18 |
| respiratory system development | GO:0060541 | 7.11E-07 | 0.00016 | 18 | BP | TNC/GRHL2/ADAMTS2/ESRP2/CRISPLD2/EYA1/DLX5/RDH10/DPPA4/FGFRL1/  MYCN/STRA6/PKDCC/PITX2/RARG/HEG1/ALDH1A3/PDGFA |
| establishment of protein localization to endoplasmic reticulum | GO:0072599 | 7.57E-07 | 0.00016 | 13 | BP | RPL31/RPL24/RPS6/RPS11/RPL32/RPL9/RPS14/RPL27A/RPS7/RPL35A/RPL12/  RPL39/RPS18 |
| axonogenesis | GO:0007409 | 7.98E-07 | 0.00016 | 28 | BP | FSTL4/NGFR/SEMA3A/MEGF8/DLX5/LHX2/CXCL12/SEMA5A/RPL24/SPP1/  FLRT3/STXBP1/TUBB2B/SSH2/CRABP2/LHX9/CNTN4/NCAM1/POU4F1/  DPYSL5/ADCY1/EFNA1/SSH3/NRXN1/CSF1R/SLITRK6/CCK/RELN |
| urogenital system development | GO:0001655 | 9.19E-07 | 0.000174 | 23 | BP | DCN/TNC/FOXC1/TP63/ITGA8/TP73/EYA1/ZBTB16/NID1/EPCAM/RDH10/  PODXL/SERPINF1/TFAP2A/STRA6/WNT9B/DACT2/HAS2/RARG/IRX3/PBX1/  PDGFA/MMP17 |
| protein localization to endoplasmic reticulum | GO:0070972 | 1.11E-06 | 0.000199 | 14 | BP | RPL31/GRIK5/RPL24/RPS6/RPS11/RPL32/RPL9/RPS14/RPL27A/RPS7/  RPL35A/RPL12/RPL39/RPS18 |
| axon development | GO:0061564 | 1.33E-06 | 0.000227 | 29 | BP | TNC/FSTL4/NGFR/SEMA3A/MEGF8/DLX5/LHX2/CXCL12/SEMA5A/RPL24/  SPP1/FLRT3/STXBP1/TUBB2B/SSH2/CRABP2/LHX9/CNTN4/NCAM1/  POU4F1/DPYSL5/ADCY1/EFNA1/SSH3/NRXN1/CSF1R/SLITRK6/CCK/RELN |
| renal system development | GO:0072001 | 1.65E-06 | 0.00027 | 21 | BP | DCN/FOXC1/TP63/ITGA8/TP73/EYA1/ZBTB16/NID1/EPCAM/RDH10/PODXL/  SERPINF1/TFAP2A/STRA6/WNT9B/DACT2/HAS2/IRX3/PBX1/PDGFA/MMP17 |
| reproductive structure development | GO:0048608 | 1.85E-06 | 0.000289 | 27 | BP | DCN/TNC/FOXC1/PRDM1/DLX3/VASH1/TP63/SEMA3A/GRHL2/COL9A3/SPP1/  RDH10/SERPINF1/TBX3/CTSV/RPS6/STRA6/LHX9/KIT/WNT9B/EOMES/PITX2/  HTRA1/IGF2/RARG/IRX5/ZFP42 |
| reproductive system development | GO:0061458 | 2.11E-06 | 0.000317 | 27 | BP | DCN/TNC/FOXC1/PRDM1/DLX3/VASH1/TP63/SEMA3A/GRHL2/COL9A3/SPP1/  RDH10/SERPINF1/TBX3/CTSV/RPS6/STRA6/LHX9/KIT/WNT9B/EOMES/PITX2/  HTRA1/IGF2/RARG/IRX5/ZFP42 |
| kidney development | GO:0001822 | 2.46E-06 | 0.000355 | 20 | BP | DCN/FOXC1/ITGA8/TP73/EYA1/ZBTB16/NID1/EPCAM/RDH10/PODXL/  SERPINF1/TFAP2A/STRA6/WNT9B/DACT2/HAS2/IRX3/PBX1/PDGFA/MMP17 |
| nuclear-transcribed mRNA catabolic process, nonsense-mediated decay | GO:0000184 | 3.22E-06 | 0.000446 | 13 | BP | RPL31/RPL24/RPS6/RPS11/RPL32/RPL9/RPS14/RPL27A/RPS7/RPL35A/RPL12/  RPL39/RPS18 |
| axon guidance | GO:0007411 | 5.77E-06 | 0.000748 | 18 | BP | NGFR/SEMA3A/MEGF8/DLX5/LHX2/CXCL12/SEMA5A/RPL24/FLRT3/TUBB2B/  LHX9/CNTN4/NCAM1/DPYSL5/EFNA1/NRXN1/CSF1R/RELN |
| ear development | GO:0043583 | 5.82E-06 | 0.000748 | 17 | BP | DLX6/MCOLN3/ITGA8/EYA1/HPN/DLX5/PRRX1/RDH10/MYCN/TFAP2A/MYO7A/  STRA6/CXCL14/FREM2/CTHRC1/ALDH1A3/SLITRK6 |
| neuron projection guidance | GO:0097485 | 6.52E-06 | 0.000809 | 18 | BP | NGFR/SEMA3A/MEGF8/DLX5/LHX2/CXCL12/SEMA5A/RPL24/FLRT3/TUBB2B/  LHX9/CNTN4/NCAM1/DPYSL5/EFNA1/NRXN1/CSF1R/RELN |
| embryonic skeletal system morphogenesis | GO:0048704 | 7.50E-06 | 0.000901 | 11 | BP | GRHL2/EYA1/MEGF8/PRRX1/RDH10/MYCN/TFAP2A/WNT9A/WNT9B/HOXB2/  IRX5 |
| morphogenesis of a branching structure | GO:0001763 | 8.46E-06 | 0.000983 | 16 | BP | TNC/PRDM1/TP63/SEMA3A/GRHL2/ESRP2/EYA1/RDH10/MYCN/TBX3/  WNT9B/PITX2/SEMA3E/PBX1/PDGFA/TDGF1 |
| viral transcription | GO:0019083 | 1.06E-05 | 0.001195 | 15 | BP | RPL31/HPN/RPL24/RPS6/RPS11/RPL32/RPL9/RPS14/RPL27A/TRIM8/RPS7/  RPL35A/RPL12/RPL39/RPS18 |
| inner ear morphogenesis | GO:0042472 | 1.14E-05 | 0.001245 | 11 | BP | DLX6/ITGA8/EYA1/HPN/DLX5/PRRX1/TFAP2A/MYO7A/CTHRC1/ALDH1A3/  SLITRK6 |
| face development | GO:0060324 | 1.44E-05 | 0.001522 | 8 | BP | GRHL2/CRISPLD2/DLX5/DKK1/SOX3/STRA6/RARG/ALDH1A3 |
| gland development | GO:0048732 | 1.55E-05 | 0.001562 | 25 | BP | CPS1/CDH1/TNC/FOXC1/PKM/PITX1/TP63/SEMA3A/ESRP2/HPN/IRF6/  SERPINF1/SOX3/TBX3/STRA6/RPL32/PITX2/ITGA2/STAT6/RARG/CSF1R/  ALDH1A3/PBX1/PDGFA/TDGF1 |
| tube morphogenesis | GO:0035239 | 1.56E-05 | 0.001562 | 22 | BP | TNC/TP63/GRHL2/ESRP2/EYA1/MEGF8/LHX2/RDH10/PODXL/MYCN/TBX3/  STRA6/WNT9B/FOXH1/PITX2/CTHRC1/C2CD3/SEMA3E/RARG/IRX3/CSF1R/  PBX1 |
| sensory organ morphogenesis | GO:0090596 | 1.83E-05 | 0.001731 | 18 | BP | DLX6/PRDM1/ITGA8/EYA1/HPN/DLX5/PRRX1/TFAP2A/MYO7A/STRA6/  WNT9A/MEIS1/PITX2/CTHRC1/RARG/IRX5/ALDH1A3/SLITRK6 |
| embryonic camera-type eye development | GO:0031076 | 1.83E-05 | 0.001731 | 7 | BP | RDH10/NES/TFAP2A/STRA6/PITX2/RARG/ALDH1A3 |
| embryonic skeletal system development | GO:0048706 | 1.91E-05 | 0.001759 | 12 | BP | GRHL2/EYA1/MEGF8/PRRX1/RDH10/MYCN/TFAP2A/WNT9A/WNT9B/  HOXB2/IRX5/PBX1 |
| inner ear development | GO:0048839 | 1.95E-05 | 0.001759 | 15 | BP | DLX6/MCOLN3/ITGA8/EYA1/HPN/DLX5/PRRX1/MYCN/TFAP2A/MYO7A/  CXCL14/FREM2/CTHRC1/ALDH1A3/SLITRK6 |
| protein targeting to membrane | GO:0006612 | 2.47E-05 | 0.002173 | 14 | BP | RPL31/RPL24/RPS6/RPS11/RPL32/STOM/RPL9/RPS14/RPL27A/RPS7/  RPL35A/RPL12/RPL39/RPS18 |
| viral gene expression | GO:0019080 | 2.69E-05 | 0.00231 | 15 | BP | RPL31/HPN/RPL24/RPS6/RPS11/RPL32/RPL9/RPS14/RPL27A/TRIM8/  RPS7/RPL35A/RPL12/RPL39/RPS18 |
| epithelial tube morphogenesis | GO:0060562 | 2.91E-05 | 0.002433 | 20 | BP | TNC/GRHL2/ESRP2/EYA1/MEGF8/LHX2/RDH10/PODXL/MYCN/TBX3/  WNT9B/FOXH1/PITX2/CTHRC1/C2CD3/SEMA3E/RARG/IRX3/CSF1R/PBX1 |
| forelimb morphogenesis | GO:0035136 | 3.12E-05 | 0.002552 | 7 | BP | TP63/ZBTB16/RDH10/TBX3/TFAP2A/CRABP2/WNT9A |
| digestive tract development | GO:0048565 | 3.67E-05 | 0.002938 | 12 | BP | CPS1/PRDM1/TP63/MEGF8/RARRES2/STRA6/PDGFC/KIT/PKDCC/PITX2/  CLMP/MIXL1 |
| extracellular matrix disassembly | GO:0022617 | 4.09E-05 | 0.003206 | 10 | BP | DCN/TLL1/CDH1/HPN/NID1/SPP1/CTSV/CARMIL2/FGFR4/HTRA1 |
| lung development | GO:0030324 | 4.22E-05 | 0.00323 | 14 | BP | TNC/GRHL2/ADAMTS2/ESRP2/CRISPLD2/EYA1/RDH10/DPPA4/MYCN/  STRA6/PKDCC/PITX2/HEG1/PDGFA |
| embryonic hindlimb morphogenesis | GO:0035116 | 4.31E-05 | 0.003237 | 6 | BP | PITX1/TP63/ZBTB16/TBX3/PITX2/RARG |
| respiratory tube development | GO:0030323 | 5.43E-05 | 0.003993 | 14 | BP | TNC/GRHL2/ADAMTS2/ESRP2/CRISPLD2/EYA1/RDH10/DPPA4/MYCN/  STRA6/PKDCC/PITX2/HEG1/PDGFA |
| diencephalon development | GO:0021536 | 5.99E-05 | 0.004312 | 9 | BP | CDH1/PITX1/SEMA3A/SEMA5A/KCNC1/SOX3/POU4F1/PITX2/CNTNAP2 |
| morphogenesis of a branching epithelium | GO:0061138 | 6.15E-05 | 0.004342 | 14 | BP | TNC/TP63/SEMA3A/GRHL2/ESRP2/EYA1/RDH10/MYCN/TBX3/WNT9B/  PITX2/SEMA3E/PBX1/PDGFA |
| ear morphogenesis | GO:0042471 | 6.37E-05 | 0.004411 | 11 | BP | DLX6/ITGA8/EYA1/HPN/DLX5/PRRX1/TFAP2A/MYO7A/CTHRC1/ALDH1A3/  SLITRK6 |
| establishment of protein localization to membrane | GO:0090150 | 6.79E-05 | 0.004613 | 18 | BP | RPL31/TP63/TP73/RPL24/RPS6/RPS11/RPL32/STOM/PKDCC/RPL9/RPS14/  RPL27A/NSG1/RPS7/RPL35A/RPL12/RPL39/RPS18 |
| embryonic forelimb morphogenesis | GO:0035115 | 7.76E-05 | 0.005178 | 6 | BP | TP63/RDH10/TBX3/TFAP2A/CRABP2/WNT9A |
| heart development | GO:0007507 | 8.94E-05 | 0.005833 | 26 | BP | FOXC1/PRDM1/TP73/GRHL2/EYA1/MEGF8/DKK1/FLRT3/FGFRL1/TBX3/  STRA6/MEIS1/FREM2/POU4F1/ACTC1/FOXH1/EOMES/PITX2/C2CD3/  ARMC4/EFNA1/HAS2/HEG1/SLC8A1/MIXL1/TDGF1 |
| response to mechanical stimulus | GO:0009612 | 9.21E-05 | 0.005833 | 15 | BP | DCN/TNC/HPN/CXCL12/KCNJ2/KCNC1/STRA6/ACTA1/KIT/ITGA2/MYD88/  CNTNAP2/NRXN1/SLC8A1/SLITRK6 |
| regulation of axon guidance | GO:1902667 | 9.41E-05 | 0.005833 | 5 | BP | SEMA3A/MEGF8/CXCL12/SEMA5A/TUBB2B |
| digestive system development | GO:0055123 | 9.53E-05 | 0.005833 | 12 | BP | CPS1/PRDM1/TP63/MEGF8/RARRES2/STRA6/PDGFC/KIT/PKDCC/PITX2/  CLMP/MIXL1 |
| forebrain development | GO:0030900 | 9.58E-05 | 0.005833 | 21 | BP | CDH1/PITX1/SEMA3A/DLX5/LHX2/CXCL12/DKK1/SEMA5A/KCNC1/SOX3/  TBX3/SLC4A10/POU4F1/EOMES/PITX2/CNTNAP2/CSF1R/SLC8A1/PCDH9/  ALDH1A3/RELN |
| skeletal system morphogenesis | GO:0048705 | 9.72E-05 | 0.005833 | 15 | BP | FOXC1/GRHL2/EYA1/MEGF8/DLX5/PRRX1/RDH10/MYCN/TFAP2A/WNT9A/  WNT9B/HAS2/RARG/HOXB2/IRX5 |
| cranial skeletal system development | GO:1904888 | 0.000104 | 0.006167 | 8 | BP | TP63/GRHL2/MEGF8/PRRX1/RDH10/TFAP2A/WNT9B/IRX5 |
| cell-cell adhesion via plasma-membrane adhesion molecules | GO:0098742 | 0.000113 | 0.006558 | 16 | BP | CDH1/CDH3/CDH7/CBLN1/EPCAM/FLRT3/FGFRL1/DSG3/PCDH10/CELSR2/  MPZL2/CLDN3/SMAGP/CDH5/NRXN1/PCDH9 |
| positive regulation of cellular component movement | GO:0051272 | 0.000116 | 0.006644 | 24 | BP | CD74/SEMA3A/MEGF8/RARRES2/CXCL12/GCNT2/SEMA5A/EPCAM/PODXL/  RAB25/TUBB2B/SEMA6D/PDGFC/CXCL14/KIT/CARMIL2/ITGA2/HAS2/P2RY6/  CSF1R/SLC8A1/RELN/PDGFA/TDGF1 |
| translational initiation | GO:0006413 | 0.000132 | 0.007401 | 14 | BP | RPL31/RPL24/RPS6/RPS11/RPL32/RPL9/RPS14/RPL27A/RPS7/RPL35A/  RPL12/RPL39/EIF3CL/RPS18 |
| negative chemotaxis | GO:0050919 | 0.000154 | 0.008556 | 6 | BP | SEMA3A/SEMA5A/FLRT3/SEMA3D/SEMA3E/PDGFA |
| auditory behavior | GO:0031223 | 0.000167 | 0.009109 | 4 | BP | STRA6/CNTNAP2/NRXN1/SLITRK6 |
| hindlimb morphogenesis | GO:0035137 | 0.000181 | 0.009717 | 6 | BP | PITX1/TP63/ZBTB16/TBX3/PITX2/RARG |
| somatic stem cell population maintenance | GO:0035019 | 0.000198 | 0.010468 | 8 | BP | TP63/DPPA4/ZSCAN10/KIT/WNT9B/PBX1/POU5F1/TDGF1 |
| stem cell population maintenance | GO:0019827 | 0.000208 | 0.010691 | 12 | BP | TP63/PRRX1/DPPA4/ZSCAN10/TBX3/KIT/WNT9B/EOMES/TRIM8/  PBX1/POU5F1/TDGF1 |
| regulation of axonogenesis | GO:0050770 | 0.000208 | 0.010691 | 12 | BP | FSTL4/NGFR/SEMA3A/MEGF8/CXCL12/SEMA5A/SPP1/TUBB2B/  SSH2/CRABP2/EFNA1/SSH3 |
| positive regulation of locomotion | GO:0040017 | 0.000217 | 0.010831 | 24 | BP | CD74/SEMA3A/MEGF8/RARRES2/CXCL12/GCNT2/SEMA5A/EPCAM/  PODXL/RAB25/TUBB2B/SEMA6D/PDGFC/CXCL14/KIT/CARMIL2/ITGA2/  HAS2/P2RY6/CSF1R/SLC8A1/RELN/PDGFA/TDGF1 |
| mesoderm morphogenesis | GO:0048332 | 0.000218 | 0.010831 | 8 | BP | FOXC1/EYA2/ITGA8/EYA1/DKK1/TBX3/EOMES/ITGA2 |
| tissue migration | GO:0090130 | 0.00022 | 0.010831 | 16 | BP | DCN/VASH1/SEMA3A/GRHL2/FERMT1/SEMA5A/SERPINF1/RAB25/ACTA1/  KIT/ACTC1/ITGA2/EFNA1/HAS2/PLEKHG5/TDGF1 |
| response to auditory stimulus | GO:0010996 | 0.000228 | 0.01108 | 5 | BP | KCNC1/STRA6/CNTNAP2/NRXN1/SLITRK6 |
| regionalization | GO:0003002 | 0.000232 | 0.011122 | 19 | BP | FOXC1/TP63/SEMA3A/MEGF8/LHX2/DKK1/ZBTB16/TBX3/CELSR2/FOXH1/  EOMES/PITX2/SP8/RARG/HOXB2/IRX3/PBX1/RELN/TDGF1 |
| maintenance of cell number | GO:0098727 | 0.000235 | 0.011122 | 12 | BP | TP63/PRRX1/DPPA4/ZSCAN10/TBX3/KIT/WNT9B/EOMES/TRIM8/PBX1/  POU5F1/TDGF1 |
| muscle tissue development | GO:0060537 | 0.000248 | 0.011509 | 20 | BP | DCN/FOXC1/EYA2/PITX1/TP63/ITGA8/TP73/EYA1/DKK1/TBX3/STRA6/  ACTA1/MEIS1/POU4F1/ACTC1/FOXH1/EOMES/PITX2/HEG1/SLC8A1 |
| developmental growth involved in morphogenesis | GO:0060560 | 0.000249 | 0.011509 | 14 | BP | TNC/FSTL4/CYFIP2/GAL/SEMA3A/MEGF8/LHX2/CXCL12/SEMA5A/SPP1/  RDH10/FLRT3/CRABP2/RARG |
| nerve development | GO:0021675 | 0.000265 | 0.011998 | 8 | BP | SEMA3A/RPL24/CTSV/TFAP2A/POU4F1/HOXB2/SLITRK6/UNC13B |
| heart morphogenesis | GO:0003007 | 0.000267 | 0.011998 | 15 | BP | FOXC1/GRHL2/EYA1/MEGF8/DKK1/FGFRL1/TBX3/POU4F1/ACTC1/  FOXH1/PITX2/C2CD3/EFNA1/HAS2/HEG1 |
| pattern specification process | GO:0007389 | 0.000294 | 0.013082 | 22 | BP | FOXC1/TP63/SEMA3A/EYA1/MEGF8/LHX2/DKK1/ZBTB16/TBX3/CELSR2/  FOXH1/EOMES/PITX2/SP8/C2CD3/ARMC4/RARG/HOXB2/IRX3/PBX1/  RELN/TDGF1 |
| mechanosensory behavior | GO:0007638 | 0.000307 | 0.013496 | 4 | BP | STRA6/CNTNAP2/NRXN1/SLITRK6 |
| cardiac ventricle development | GO:0003231 | 0.000344 | 0.014945 | 10 | BP | FOXC1/PRDM1/GRHL2/FGFRL1/TBX3/STRA6/POU4F1/FOXH1/PITX2/  HEG1 |
| eye development | GO:0001654 | 0.000364 | 0.015619 | 19 | BP | FOXC1/PRDM1/GRHL2/LHX2/RPL24/RDH10/SERPINF1/NES/TFAP2A/  MYO7A/STRA6/WNT9A/MEIS1/WNT9B/PITX2/RARG/IRX5/ALDH1A3/SLITRK6 |
| regulation of cell morphogenesis | GO:0022604 | 0.000369 | 0.015648 | 22 | BP | FSTL4/NGFR/SEMA3A/FBLN1/BAMBI/MEGF8/HPN/CXCL12/SEMA5A/SPP1/  TUBB2B/SSH2/CRABP2/TTBK1/EPS8/KIT/EFNA1/SEMA3E/HAS2/SSH3/  CSF1R/RELN |
| synaptic transmission, glutamatergic | GO:0035249 | 0.000383 | 0.016048 | 8 | BP | GRIK5/DKK1/GRM4/STXBP1/GRIK3/NRXN1/RELN/UNC13B |
| neural nucleus development | GO:0048857 | 0.000392 | 0.016238 | 7 | BP | KCNC1/TTBK1/INA/PITX2/CKB/HOXB2/ALDH1A3 |
| nose development | GO:0043584 | 0.000402 | 0.01628 | 4 | BP | DLX5/RDH10/STRA6/ALDH1A3 |
| regulation of axon extension involved in axon guidance | GO:0048841 | 0.000402 | 0.01628 | 4 | BP | SEMA3A/MEGF8/CXCL12/SEMA5A |
| gland morphogenesis | GO:0022612 | 0.000423 | 0.016917 | 10 | BP | TNC/TP63/SEMA3A/ESRP2/HPN/TBX3/STAT6/RARG/CSF1R/PDGFA |
| mesenchyme development | GO:0060485 | 0.00044 | 0.017412 | 15 | BP | FOXC1/SEMA3A/BAMBI/HPN/GCNT2/RDH10/DPPA4/ACTA1/ACTC1/  FOXH1/EOMES/PITX2/EFNA1/HAS2/BNC2 |
| regulation of epidermis development | GO:0045682 | 0.000457 | 0.017874 | 8 | BP | FOXC1/CDH3/GAL/TP63/GRHL2/FERMT1/MYCN/CTSV |
| embryonic cranial skeleton morphogenesis | GO:0048701 | 0.000479 | 0.018518 | 6 | BP | GRHL2/PRRX1/RDH10/TFAP2A/WNT9B/IRX5 |
| camera-type eye development | GO:0043010 | 0.000483 | 0.018518 | 17 | BP | FOXC1/GRHL2/LHX2/RPL24/RDH10/SERPINF1/NES/TFAP2A/STRA6/  WNT9A/MEIS1/WNT9B/PITX2/RARG/IRX5/ALDH1A3/SLITRK6 |
| negative regulation of epidermis development | GO:0045683 | 0.000517 | 0.019557 | 4 | BP | CDH3/TP63/GRHL2/FERMT1 |
| positive regulation of cell motility | GO:2000147 | 0.000521 | 0.019557 | 22 | BP | CD74/SEMA3A/RARRES2/CXCL12/GCNT2/SEMA5A/EPCAM/PODXL/  RAB25/SEMA6D/PDGFC/CXCL14/KIT/CARMIL2/ITGA2/HAS2/P2RY6/  CSF1R/SLC8A1/RELN/PDGFA/TDGF1 |
| negative regulation of neuron differentiation | GO:0045665 | 0.00054 | 0.019814 | 13 | BP | FSTL4/NGFR/SEMA3A/TP73/SEMA5A/SPP1/SOX3/MEIS1/CNTN4/FUOM/  EFNA1/IRX3/PBX1 |
| positive regulation of ossification | GO:0045778 | 0.000541 | 0.019814 | 8 | BP | TP63/ZBTB16/TFAP2A/PKDCC/CTHRC1/NELL1/ZHX3/SLC8A1 |
| epithelial cell morphogenesis | GO:0003382 | 0.000556 | 0.019814 | 5 | BP | GRHL2/RAB25/DACT2/CLDN3/HEG1 |
| olfactory bulb development | GO:0021772 | 0.000556 | 0.019814 | 5 | BP | SEMA3A/DLX5/LHX2/EOMES/CSF1R |
| relaxation of muscle | GO:0090075 | 0.000556 | 0.019814 | 5 | BP | PDE4D/KCNJ2/GUCY1A3/SLN/SLC8A1 |
| placenta development | GO:0001890 | 0.000564 | 0.019821 | 11 | BP | DCN/PRDM1/DLX3/VASH1/GRHL2/SPP1/CTSV/RPS6/EOMES/HTRA1/  IGF2 |
| epithelial cell development | GO:0002064 | 0.000567 | 0.019821 | 13 | BP | PRDM1/TP63/GRHL2/PDE4D/PODXL/RAB25/MYO7A/DACT2/CLDN3/  RARG/HEG1/CDH5/SLITRK6 |
| ameboidal-type cell migration | GO:0001667 | 0.000618 | 0.021385 | 18 | BP | DCN/VASH1/SEMA3A/FERMT1/MEGF8/SEMA5A/SERPINF1/RAB25/  KIT/PITX2/ITGA2/EFNA1/HAS2/PLEKHG5/C5orf30/SLC8A1/MIXL1/TDGF1 |
| olfactory lobe development | GO:0021988 | 0.00065 | 0.02216 | 5 | BP | SEMA3A/DLX5/LHX2/EOMES/CSF1R |
| positive regulation of protein depolymerization | GO:1901881 | 0.000652 | 0.02216 | 4 | BP | SEMA5A/NES/CARMIL2/SLN |
| positive regulation of cell migration | GO:0030335 | 0.000735 | 0.024743 | 21 | BP | CD74/SEMA3A/RARRES2/CXCL12/GCNT2/SEMA5A/PODXL/RAB25/  SEMA6D/PDGFC/CXCL14/KIT/CARMIL2/ITGA2/HAS2/P2RY6/CSF1R/  SLC8A1/RELN/PDGFA/TDGF1 |
| neuron recognition | GO:0008038 | 0.000756 | 0.024984 | 5 | BP | SEMA3A/MEGF8/SEMA5A/CNTN4/CNTNAP2 |
| mesodermal cell differentiation | GO:0048333 | 0.000756 | 0.024984 | 5 | BP | EYA2/ITGA8/EYA1/DKK1/ITGA2 |
| regulation of cell morphogenesis involved in differentiation | GO:0010769 | 0.00086 | 0.02773 | 15 | BP | FSTL4/NGFR/SEMA3A/FBLN1/MEGF8/CXCL12/SEMA5A/SPP1/TUBB2B/  SSH2/CRABP2/EFNA1/HAS2/SSH3/RELN |
| negative regulation of cell development | GO:0010721 | 0.00086 | 0.02773 | 16 | BP | FSTL4/NGFR/GAL/SEMA3A/FBLN1/TP73/SEMA5A/SPP1/MYCN/SOX3/  MEIS1/CNTN4/FUOM/EFNA1/IRX3/PBX1 |
| cranial nerve development | GO:0021545 | 0.000862 | 0.02773 | 6 | BP | SEMA3A/RPL24/TFAP2A/POU4F1/HOXB2/SLITRK6 |
| nuclear-transcribed mRNA catabolic process | GO:0000956 | 0.000906 | 0.02887 | 13 | BP | RPL31/RPL24/RPS6/RPS11/RPL32/RPL9/RPS14/RPL27A/RPS7/RPL35A/  RPL12/RPL39/RPS18 |
| axonal fasciculation | GO:0007413 | 0.000996 | 0.030974 | 4 | BP | SEMA3A/MEGF8/SEMA5A/CNTN4 |
| neuron projection fasciculation | GO:0106030 | 0.000996 | 0.030974 | 4 | BP | SEMA3A/MEGF8/SEMA5A/CNTN4 |
| epithelial cell proliferation | GO:0050673 | 0.000999 | 0.030974 | 18 | BP | DLX6/CDH3/NGFR/VASH1/TP63/FERMT1/ESRP2/EYA1/HPN/DLX5/  CXCL12/SEMA5A/IRF6/SERPINF1/KIT/HTRA1/STAT6/HAS2 |
| cell fate commitment involved in formation of primary germ layer | GO:0060795 | 0.001006 | 0.030974 | 5 | BP | EYA2/EYA1/DKK1/EOMES/POU5F1 |
| mesoderm formation | GO:0001707 | 0.00102 | 0.031118 | 7 | BP | FOXC1/EYA2/ITGA8/EYA1/DKK1/EOMES/ITGA2 |
| female sex differentiation | GO:0046660 | 0.001193 | 0.035907 | 9 | BP | FOXC1/TP63/COL9A3/TBX3/STRA6/LHX9/KIT/PITX2/ZFP42 |
| axon extension involved in axon guidance | GO:0048846 | 0.001208 | 0.035907 | 4 | BP | SEMA3A/MEGF8/CXCL12/SEMA5A |
| neuron projection extension involved in neuron projection guidance | GO:1902284 | 0.001208 | 0.035907 | 4 | BP | SEMA3A/MEGF8/CXCL12/SEMA5A |
| negative regulation of cell adhesion | GO:0007162 | 0.001217 | 0.035907 | 14 | BP | CD74/CDH1/TNC/FBLN1/CXCL12/GCNT2/SEMA5A/EPCAM/TGFBI/  PODXL/ASS1/DACT2/SEMA3E/C1QTNF1 |
| palate development | GO:0060021 | 0.001259 | 0.03669 | 8 | BP | DLX6/DLX5/PRRX1/TBX3/TFAP2A/WNT9B/PKDCC/BNC2 |
| sex differentiation | GO:0007548 | 0.001266 | 0.03669 | 15 | BP | FOXC1/TP63/SEMA3A/COL9A3/RDH10/TBX3/CTSV/STRA6/LHX9/KIT/  WNT9B/PITX2/IRX5/ZFP42/PBX1 |
| diterpenoid biosynthetic process | GO:0016102 | 0.001284 | 0.03669 | 3 | BP | RDH10/CRABP2/ALDH1A3 |
| learned vocalization behavior or vocal learning | GO:0098598 | 0.001284 | 0.03669 | 3 | BP | STRA6/CNTNAP2/NRXN1 |
| developmental cell growth | GO:0048588 | 0.001392 | 0.039305 | 12 | BP | FSTL4/CYFIP2/GAL/SEMA3A/MEGF8/LHX2/CXCL12/SEMA5A/SPP1/  FLRT3/CRABP2/RARG |
| positive regulation of epithelial cell proliferation | GO:0050679 | 0.001397 | 0.039305 | 11 | BP | DLX6/CDH3/TP63/ESRP2/EYA1/HPN/DLX5/CXCL12/SEMA5A/HTRA1/HAS2 |
| cell adhesion mediated by integrin | GO:0033627 | 0.001447 | 0.040387 | 6 | BP | FERMT1/ITGB6/PODXL/ITGA2/SYK/EFNA1 |
| keratinocyte proliferation | GO:0043616 | 0.00149 | 0.041276 | 5 | BP | CDH3/TP63/FERMT1/IRF6/HAS2 |
| epithelium migration | GO:0090132 | 0.001541 | 0.042363 | 14 | BP | DCN/VASH1/SEMA3A/GRHL2/FERMT1/SEMA5A/SERPINF1/RAB25/  KIT/ITGA2/EFNA1/HAS2/PLEKHG5/TDGF1 |
| formation of primary germ layer | GO:0001704 | 0.001616 | 0.04378 | 9 | BP | FOXC1/EYA2/ITGA8/EYA1/DKK1/EOMES/ITGA2/MIXL1/POU5F1 |
| positive regulation of peptidyl-tyrosine phosphorylation | GO:0050731 | 0.001617 | 0.04378 | 11 | BP | CNTN1/CD74/KIT/SYK/IGF2/EFNA1/STAP2/CSF1R/CCK/RELN/TDGF1 |
| heart valve development | GO:0003170 | 0.001684 | 0.044919 | 5 | BP | PRDM1/FGFRL1/STRA6/PITX2/EFNA1 |
| hair cell differentiation | GO:0035315 | 0.001684 | 0.044919 | 5 | BP | MCOLN3/MYCN/MYO7A/PITX2/SLITRK6 |
| negative regulation of neurogenesis | GO:0050768 | 0.001729 | 0.04561 | 14 | BP | FSTL4/NGFR/SEMA3A/TP73/SEMA5A/SPP1/MYCN/SOX3/MEIS1/  CNTN4/FUOM/EFNA1/IRX3/PBX1 |
| biological phase | GO:0044848 | 0.001735 | 0.04561 | 3 | BP | CDH3/GAL/FERMT1 |
| eye morphogenesis | GO:0048592 | 0.001748 | 0.045619 | 10 | BP | PRDM1/TFAP2A/MYO7A/STRA6/WNT9A/MEIS1/PITX2/RARG/IRX5/  ALDH1A3 |
| epidermis development | GO:0008544 | 0.001884 | 0.048796 | 21 | BP | PRSS8/FOXC1/MCOLN3/CDH3/GAL/TP63/GRHL2/FERMT1/LHX2/  DKK1/IRF6/MYCN/DSG3/CTSV/MYO7A/CRABP2/PITX2/AGPAT2/PKP3/  SLITRK6/PDGFA |
| branching morphogenesis of an epithelial tube | GO:0048754 | 0.001938 | 0.049292 | 10 | BP | TNC/ESRP2/EYA1/RDH10/MYCN/TBX3/WNT9B/PITX2/SEMA3E/PBX1 |
| glutamate receptor signaling pathway | GO:0007215 | 0.001944 | 0.049292 | 7 | BP | NECAB2/GRIN2D/GRIK5/GRM4/CACNG8/GRIK3/RELN |
| negative regulation of developmental growth | GO:0048640 | 0.001944 | 0.049292 | 7 | BP | FSTL4/GAL/SEMA3A/TP73/SEMA5A/SPP1/MEIS1 |
| proteinaceous extracellular matrix | GO:0005578 | 1.97E-07 | 6.60E-05 | 26 | CC | DCN/TNC/FBLN1/ADAMTS2/COL9A3/CRISPLD2/NID1/TGFBI/FLRT3/  SERPINF1/ADAMTS17/COL6A2/WNT9A/HAPLN1/ADAMTS19/FREM2/  SPARCL1/WNT9B/COL6A3/CTHRC1/VWA2/EFEMP2/HPSE2/RELN/  MMP17/COL6A6 |
| cell-cell junction | GO:0005911 | 3.83E-07 | 6.60E-05 | 28 | CC | CYTH3/CDH1/CDH3/GRHL2/HPN/EPCAM/KCNJ2/FLRT3/FGFRL1/PODXL/  TRPC4/DSG3/TLN1/TRIM29/PGM5/KIT/FGFR4/S100A11/CLDN3/CLMP/  SMAGP/PLEKHG5/HEG1/CNTNAP2/CDH5/SLC8A1/PCDH9/PKP3 |
| cytosolic ribosome | GO:0022626 | 1.21E-06 | 0.000139 | 13 | CC | RPL31/RPL24/RPS6/RPS11/RPL32/RPL9/RPS14/RPL27A/RPS7/RPL35A/  RPL12/RPL39/RPS18 |
| presynapse | GO:0098793 | 1.83E-05 | 0.001575 | 22 | CC | DBNDD1/TSPOAP1/SYT7/CTTNBP2/AMPH/GRIK5/GRM4/PACSIN1/  FLRT3/NTS/MICAL1/STXBP1/SLC40A1/SYTL1/SYTL5/SYNPR/GRIK3/  ITGA2/KCTD12/NRXN1/CCK/UNC13B |
| axon terminus | GO:0043679 | 5.51E-05 | 0.003354 | 11 | CC | SYT7/GRIK5/PACSIN1/FLRT3/NTS/MICAL1/STXBP1/GRIK3/ITGA2/CCK/  UNC13B |
| cytosolic large ribosomal subunit | GO:0022625 | 5.83E-05 | 0.003354 | 8 | CC | RPL31/RPL24/RPL32/RPL9/RPL27A/RPL35A/RPL12/RPL39 |
| neuron projection terminus | GO:0044306 | 0.000168 | 0.008299 | 11 | CC | SYT7/GRIK5/PACSIN1/FLRT3/NTS/MICAL1/STXBP1/GRIK3/ITGA2/CCK/  UNC13B |
| ribosomal subunit | GO:0044391 | 0.000236 | 0.010192 | 13 | CC | RPL31/RPL24/RPS6/RPS11/RPL32/RPL9/RPS14/RPL27A/RPS7/RPL35A/  RPL12/RPL39/RPS18 |
| cytosolic part | GO:0044445 | 0.000297 | 0.011373 | 15 | CC | RPL31/RPL24/RPS6/RPS11/RPL32/RPL9/GUCY1A3/RPS14/ADCY1/  RPL27A/RPS7/RPL35A/RPL12/RPL39/RPS18 |
| terminal bouton | GO:0043195 | 0.000437 | 0.015085 | 7 | CC | SYT7/GRIK5/MICAL1/STXBP1/GRIK3/CCK/UNC13B |
| focal adhesion | GO:0005925 | 0.000644 | 0.018789 | 20 | CC | TNC/TLE2/RPL31/ITGA8/FERMT1/ITGB6/FLRT3/FLNC/AKAP12/LMO7/  TLN1/RPS11/PGM5/ACTC1/RPL9/ITGA2/RPS14/RPS7/RPL12/RPS18 |
| anchored component of membrane | GO:0031225 | 0.000691 | 0.018789 | 11 | CC | PRSS22/CNTN1/PRSS8/BST2/CNTN4/NCAM1/CNTN5/LYPD6B/EFNA1/  MMP17/TDGF1 |
| cell-substrate adherens junction | GO:0005924 | 0.000708 | 0.018789 | 20 | CC | TNC/TLE2/RPL31/ITGA8/FERMT1/ITGB6/FLRT3/FLNC/AKAP12/LMO7/  TLN1/RPS11/PGM5/ACTC1/RPL9/ITGA2/RPS14/RPS7/RPL12/RPS18 |
| cell-substrate junction | GO:0030055 | 0.000802 | 0.019468 | 20 | CC | TNC/TLE2/RPL31/ITGA8/FERMT1/ITGB6/FLRT3/FLNC/AKAP12/LMO7/  TLN1/RPS11/PGM5/ACTC1/RPL9/ITGA2/RPS14/RPS7/RPL12/RPS18 |
| cell-cell adherens junction | GO:0005913 | 0.000846 | 0.019468 | 8 | CC | CDH1/CDH3/TRIM29/PGM5/S100A11/SMAGP/CDH5/PKP3 |
| cell body | GO:0044297 | 0.000941 | 0.019547 | 23 | CC | SYT7/GAL/ITGA8/HPN/GRIK5/RDH10/KCNJ2/PODXL/KCNC1/SERPINF1/  CTSV/RPS6/ACTA1/SLC4A10/ASTN1/DPYSL5/ACTC1/GRIK3/CKB/  CNTNAP2/NRXN1/KCND2/CCK |
| synaptic membrane | GO:0097060 | 0.001011 | 0.019547 | 16 | CC | SYT7/ITGA8/CBLN1/GRIN2D/GRIK5/GRM4/FLRT3/LRRC4/STXBP1/  CACNG8/GRIK3/NSG1/KCTD12/NRXN1/SLC8A1/KCND2 |
| cation channel complex | GO:0034703 | 0.00102 | 0.019547 | 13 | CC | KCNK6/GRIN2D/GRIK5/PDE4D/KCNJ2/KCNC1/TRPC4/CACNG8/EPS8/  CACNA1D/CNTNAP2/KCND2/KCNQ5 |
| basement membrane | GO:0005604 | 0.001134 | 0.020586 | 8 | CC | TNC/FBLN1/NID1/TGFBI/SERPINF1/FREM2/VWA2/EFEMP2 |
| ribosome | GO:0005840 | 0.002131 | 0.031758 | 13 | CC | RPL31/RPL24/RPS6/RPS11/RPL32/RPL9/RPS14/RPL27A/RPS7/RPL35A/  RPL12/RPL39/RPS18 |
| side of membrane | GO:0098552 | 0.002186 | 0.031758 | 20 | CC | MATK/CYTH3/CD74/CDH1/CXCL12/ITGB6/TRAF3/CTSV/NCAM1/  ASTN1/PGM5/KIT/SHROOM4/CARMIL2/BDH1/ITGA2/SYK/HEG1/  CDH5/BCAM |
| ion channel complex | GO:0034702 | 0.002202 | 0.031758 | 15 | CC | CFTR/KCNK6/GRIN2D/GRIK5/PDE4D/KCNJ2/KCNC1/TRPC4/CACNG8/  EPS8/CACNA1D/LRRC8C/CNTNAP2/KCND2/KCNQ5 |
| microvillus | GO:0005902 | 0.002208 | 0.031758 | 7 | CC | PODXL/CTSV/MYO7A/SYTL1/PROM2/FMN2/PDGFA |
| extrinsic component of plasma membrane | GO:0019897 | 0.002209 | 0.031758 | 10 | CC | PRSS22/MATK/CYTH3/CDH1/EPN3/PRSS8/SYTL1/CARMIL2/SYK/TDGF1 |
| axon part | GO:0033267 | 0.002577 | 0.035566 | 11 | CC | SYT7/GRIK5/FLRT3/KCNC1/SERPINF1/MICAL1/STXBP1/GRIK3/  CNTNAP2/CCK/UNC13B |
| cytosolic small ribosomal subunit | GO:0022627 | 0.002738 | 0.036336 | 5 | CC | RPS6/RPS11/RPS14/RPS7/RPS18 |
| actin-based cell projection | GO:0098858 | 0.003461 | 0.044219 | 11 | CC | ACPP/PODXL/CTSV/MYO7A/SYTL1/ACTA1/EPS8/PROM2/FMN2/  ACTC1/PDGFA |
| collagen trimer | GO:0005581 | 0.003786 | 0.046116 | 7 | CC | DCN/COL9A3/COL6A2/COL6A3/CTHRC1/C1QTNF1/COL6A6 |
| dendrite | GO:0030425 | 0.004018 | 0.046116 | 21 | CC | SYT7/TP63/SEMA3A/CTTNBP2/ITGA8/NECAB2/GRIK5/KCNJ2/LRRC4/  KCNC1/RPS6/SLC4A10/DPYSL5/GRIK3/CKB/NSG1/CNTNAP2/SLC8A1/  KCND2/CCK/RELN |
| cell leading edge | GO:0031252 | 0.004105 | 0.046116 | 17 | CC | CYTH3/CDH1/ITGA8/AMPH/FERMT1/PACSIN1/PODXL/KCNC1/EPS8L1/  TLN1/ACTA1/EPS8/ACTC1/CARMIL2/S100A11/PLEKHG5/CNTNAP2 |
| large ribosomal subunit | GO:0015934 | 0.004144 | 0.046116 | 8 | CC | RPL31/RPL24/RPL32/RPL9/RPL27A/RPL35A/RPL12/RPL39 |
| perikaryon | GO:0043204 | 0.004371 | 0.047125 | 8 | CC | ITGA8/GRIK5/CTSV/ASTN1/GRIK3/CNTNAP2/KCND2/CCK |
| semaphorin receptor binding | GO:0030215 | 5.15E-05 | 0.01622 | 4 | MF | SEMA3A/SEMA5A/SEMA6D/SEMA3E |
| structural constituent of ribosome | GO:0003735 | 5.65E-05 | 0.01622 | 13 | MF | RPL31/RPL24/RPS6/RPS11/RPL32/RPL9/RPS14/RPL27A/RPS7/RPL35A/  RPL12/RPL39/RPS18 |
| collagen binding | GO:0005518 | 0.000103 | 0.01678 | 8 | MF | DCN/PCOLCE/NID1/TGFBI/SPARCL1/ITGA2/C1QTNF1/PDGFA |
| neuropilin binding | GO:0038191 | 0.000117 | 0.01678 | 4 | MF | SEMA3A/SEMA5A/SEMA3D/SEMA3E |
| extracellular matrix binding | GO:0050840 | 0.000154 | 0.017699 | 7 | MF | DCN/NID1/SPP1/TGFBI/SPARCL1/ITGA2/BCAM |
| transcriptional activator activity, RNA polymerase II transcription regulatory region sequence-specific binding | GO:0001228 | 0.000479 | 0.044795 | 21 | MF | FOXC1/DLX3/PITX1/TP63/TP73/GRHL2/DLX5/LHX2/IRF6/MYCN/  TFAP2A/CREB3L4/MEIS1/POU4F1/FOXH1/PITX2/STAT6/HOXB2/MACC1/  MIXL1/PBX1 |
| metal ion transmembrane transporter activity | GO:0046873 | 0.00063 | 0.044795 | 22 | MF | MCOLN3/ATP2B3/KCNK6/GRIN2D/HPN/GRIK5/KCNJ13/KCNJ2/KCNC1/  SLC6A11/TRPC4/SLC40A1/CACNG8/SLC4A10/CACNA1D/GRIK3/KCNK5/  TMEM37/SLC8A1/KCND2/KCNQ5/SLC30A10 |
| cadherin binding involved in cell-cell adhesion | GO:0098641 | 0.000648 | 0.044795 | 4 | MF | EPCAM/TRIM29/S100A11/PKP3 |
| potassium channel activity | GO:0005267 | 0.000742 | 0.044795 | 10 | MF | KCNK6/HPN/GRIK5/KCNJ13/KCNJ2/KCNC1/GRIK3/KCNK5/KCND2/KCNQ5 |
| ankyrin binding | GO:0030506 | 0.000806 | 0.044795 | 4 | MF | CDH1/FLNC/CACNA1D/SLC8A1 |
| cation channel activity | GO:0005261 | 0.000858 | 0.044795 | 17 | MF | MCOLN3/KCNK6/GRIN2D/HPN/GRIK5/KCNJ13/KCNJ2/KCNC1/TRPC4/  SLC40A1/CACNG8/CACNA1D/GRIK3/KCNK5/TMEM37/KCND2/KCNQ5 |

**Table 2. 1 μM PBB153**

| Description | ID | pvalue | padj | Count | Category | geneID |
| --- | --- | --- | --- | --- | --- | --- |
| embryonic organ development | GO:0048568 | 1.83E-10 | 7.08E-07 | 35 | BP | DLX6/FOXC1/PRDM1/RARB/ITGA8/GRHL2/HPN/DLX5/RARRES2/  HAND1/EFEMP1/PRRX1/ID3/RDH10/NES/ATP8A2/MYCN/TBX3/  HS6ST1/TFAP2A/MYO7A/STRA6/WNT9A/KIT/WNT9B/FOXH1/PKDCC/  EOMES/PITX2/RARG/IRX5/NKX2-6/TBX1/ALDH1A3/SLITRK6 |
| muscle tissue development | GO:0060537 | 1.56E-08 | 2.62E-05 | 29 | BP | FOXC1/CDON/EYA2/PITX1/TP63/RARB/ITGA8/NEBL/TP73/KCNK2/DKK1/  HAND1/EGR1/OBSL1/TBX3/PIM1/STRA6/ACTA1/MEIS1/EPHB1/FOXH1/  ATF3/EOMES/PITX2/NKX2-6/SLC8A1/TBX1/MAFF/DNER |
| embryonic organ morphogenesis | GO:0048562 | 2.35E-08 | 2.62E-05 | 25 | BP | DLX6/RARB/ITGA8/GRHL2/HPN/DLX5/HAND1/EFEMP1/PRRX1/RDH10/  ATP8A2/MYCN/TBX3/TFAP2A/MYO7A/STRA6/WNT9A/WNT9B/FOXH1/  PITX2/RARG/IRX5/TBX1/ALDH1A3/SLITRK6 |
| appendage development | GO:0048736 | 3.39E-08 | 2.62E-05 | 19 | BP | DLX6/PITX1/TP63/RARB/GRHL2/DLX5/DKK1/PRRX1/RDH10/MYCN/  TBX3/TFAP2A/CRABP2/WNT9A/PKDCC/PITX2/RARG/KDF1/SMOC1 |
| limb development | GO:0060173 | 3.39E-08 | 2.62E-05 | 19 | BP | DLX6/PITX1/TP63/RARB/GRHL2/DLX5/DKK1/PRRX1/RDH10/MYCN/  TBX3/TFAP2A/CRABP2/WNT9A/PKDCC/PITX2/RARG/KDF1/SMOC1 |
| axon development | GO:0061564 | 4.40E-08 | 2.83E-05 | 33 | BP | SEMA3F/TNC/FSTL4/NGFR/SEMA3A/RAB21/DLX5/PTPRZ1/CXCL12/  NR2E1/SEMA5A/SPP1/PLP1/DOK4/FLRT3/ATP8A2/CRABP2/LHX9/  CNTN4/DRD2/EPHB1/DPYSL5/ADCY1/ISLR2/SSH3/NRXN1/ADGRB1/  CSF1R/CNTN2/SLITRK6/RTN4RL1/CCK/RELN |
| forebrain development | GO:0030900 | 6.62E-08 | 2.98E-05 | 28 | BP | CDON/PITX1/SEMA3A/RARB/GSK3B/LHX5/DLX5/CXCL12/DKK1/  NR2E1/SEMA5A/SOX3/TBX3/DRD2/EOMES/PITX2/ATF5/EMX2/GNG12/  CNTNAP2/NKX2-6/CSF1R/SLC8A1/CNTN2/PCDH9/ALDH1A3/RTN4RL1/  RELN |
| embryonic limb morphogenesis | GO:0030326 | 6.93E-08 | 2.98E-05 | 16 | BP | DLX6/PITX1/TP63/RARB/GRHL2/DLX5/DKK1/PRRX1/RDH10/MYCN/  TBX3/TFAP2A/CRABP2/WNT9A/PITX2/RARG |
| embryonic appendage morphogenesis | GO:0035113 | 6.93E-08 | 2.98E-05 | 16 | BP | DLX6/PITX1/TP63/RARB/GRHL2/DLX5/DKK1/PRRX1/RDH10/MYCN/  TBX3/TFAP2A/CRABP2/WNT9A/PITX2/RARG |
| skeletal system development | GO:0001501 | 9.28E-08 | 3.17E-05 | 33 | BP | DLX6/FOXC1/PITX1/TP63/RARB/GRHL2/AES/DLX5/HAND1/EFEMP1/  PRRX1/TGFBI/RDH10/MYCN/TBX3/TFAP2A/WNT9A/MEIS1/PDGFC/  HAPLN1/CER1/HMGA2/LRRK1/KIT/WNT9B/PTH1R/PKDCC/PITX2/  HAS2/RARG/IRX5/SPNS2/TBX1 |
| appendage morphogenesis | GO:0035107 | 9.85E-08 | 3.17E-05 | 17 | BP | DLX6/PITX1/TP63/RARB/GRHL2/DLX5/DKK1/PRRX1/RDH10/MYCN/  TBX3/TFAP2A/CRABP2/WNT9A/PKDCC/PITX2/RARG |
| limb morphogenesis | GO:0035108 | 9.85E-08 | 3.17E-05 | 17 | BP | DLX6/PITX1/TP63/RARB/GRHL2/DLX5/DKK1/PRRX1/RDH10/MYCN/  TBX3/TFAP2A/CRABP2/WNT9A/PKDCC/PITX2/RARG |
| sensory organ morphogenesis | GO:0090596 | 1.85E-07 | 5.51E-05 | 22 | BP | DLX6/PRDM1/CDON/RARB/ITGA8/HPN/DLX5/EFEMP1/PRRX1/ATP8A2/  TFAP2A/MYO7A/STRA6/WNT9A/MEIS1/EPHB1/PITX2/RARG/IRX5/TBX1/  ALDH1A3/SLITRK6 |
| axonogenesis | GO:0007409 | 2.43E-07 | 6.71E-05 | 30 | BP | SEMA3F/FSTL4/NGFR/SEMA3A/RAB21/DLX5/PTPRZ1/CXCL12/NR2E1/  SEMA5A/SPP1/DOK4/FLRT3/ATP8A2/CRABP2/LHX9/CNTN4/DRD2/  EPHB1/DPYSL5/ADCY1/ISLR2/SSH3/NRXN1/ADGRB1/CSF1R/CNTN2/  SLITRK6/CCK/RELN |
| eye development | GO:0001654 | 2.90E-07 | 7.42E-05 | 26 | BP | FOXC1/PRDM1/CDON/ATP2B1/RARB/GRHL2/NR2E1/EFEMP1/RDH10/  SERPINF1/NES/ATP8A2/MEIS2/TFAP2A/MYO7A/STRA6/WNT9A/MEIS1/  EPHB1/WNT9B/PITX2/RARG/IRX5/ALDH1A3/SLITRK6/SMOC1 |
| striated muscle tissue development | GO:0014706 | 3.07E-07 | 7.42E-05 | 26 | BP | FOXC1/CDON/EYA2/PITX1/RARB/NEBL/TP73/KCNK2/DKK1/HAND1/  EGR1/OBSL1/TBX3/PIM1/ACTA1/MEIS1/EPHB1/FOXH1/ATF3/EOMES/  PITX2/NKX2-6/SLC8A1/TBX1/MAFF/DNER |
| mesoderm development | GO:0007498 | 4.19E-07 | 9.53E-05 | 15 | BP | MATK/FOXC1/EYA2/TP63/ITGA8/DKK1/HAND1/TBX3/CER1/HMGA2/  FOXH1/EOMES/ITGA2/IRX3/TBX1 |
| extracellular matrix organization | GO:0030198 | 4.97E-07 | 0.000107 | 25 | BP | TNC/FOXC1/FBLN1/ITGA8/COL5A3/ADAMTS2/COL9A3/FERMT1/  CRISPLD2/HPN/NR2E1/SPP1/TGFBI/CTSV/COL6A2/HAPLN1/CARMIL2/  ITGA2/HTRA1/HAS2/EFEMP2/HPSE2/GAS6/COL4A5/COL4A6 |
| extracellular structure organization | GO:0043062 | 5.26E-07 | 0.000107 | 25 | BP | TNC/FOXC1/FBLN1/ITGA8/COL5A3/ADAMTS2/COL9A3/FERMT1/  CRISPLD2/HPN/NR2E1/SPP1/TGFBI/CTSV/COL6A2/HAPLN1/CARMIL2/  ITGA2/HTRA1/HAS2/EFEMP2/HPSE2/GAS6/COL4A5/COL4A6 |
| limbic system development | GO:0021761 | 8.00E-07 | 0.000155 | 13 | BP | SEMA3A/GSK3B/LHX5/NR2E1/SOX3/TBX3/DRD2/PITX2/EMX2/  CNTNAP2/NKX2-6/ALDH1A3/RELN |
| telencephalon development | GO:0021537 | 1.13E-06 | 0.000208 | 20 | BP | CDON/SEMA3A/RARB/GSK3B/LHX5/DLX5/CXCL12/NR2E1/DRD2/  EOMES/ATF5/EMX2/GNG12/CNTNAP2/CSF1R/SLC8A1/CNTN2/  ALDH1A3/RTN4RL1/RELN |
| eye morphogenesis | GO:0048592 | 2.14E-06 | 0.000376 | 15 | BP | PRDM1/CDON/RARB/EFEMP1/ATP8A2/TFAP2A/MYO7A/STRA6/  WNT9A/MEIS1/EPHB1/PITX2/RARG/IRX5/ALDH1A3 |
| face development | GO:0060324 | 2.37E-06 | 0.000385 | 9 | BP | GRHL2/CRISPLD2/DLX5/DKK1/SOX3/STRA6/RARG/TBX1/ALDH1A3 |
| urogenital system development | GO:0001655 | 2.39E-06 | 0.000385 | 23 | BP | TNC/FOXC1/TP63/RARB/ITGA8/TP73/ID3/EGR1/RDH10/PODXL/  SERPINF1/TFAP2A/STRA6/CER1/WNT9B/DACT2/EMX2/HAS2/RARG/  IRX3/GCNT1/MMP17/LGR4 |
| camera-type eye development | GO:0043010 | 3.27E-06 | 0.000506 | 22 | BP | FOXC1/CDON/ATP2B1/RARB/GRHL2/NR2E1/EFEMP1/RDH10/  SERPINF1/NES/ATP8A2/TFAP2A/STRA6/WNT9A/MEIS1/EPHB1/  WNT9B/PITX2/RARG/IRX5/ALDH1A3/SLITRK6 |
| learning or memory | GO:0007611 | 3.93E-06 | 0.000571 | 19 | BP | MAPK8IP2/ITGA8/KCNK2/JPH4/PTPRZ1/DKK1/GLP1R/SLC8A2/  LRRN4/SERPINF1/MEIS2/STRA6/DRD2/KIT/ADCY1/CNTNAP2/  NRXN1/CNTN2/RELN |
| renal system development | GO:0072001 | 3.99E-06 | 0.000571 | 21 | BP | FOXC1/TP63/RARB/ITGA8/TP73/ID3/EGR1/RDH10/PODXL/SERPINF1/  TFAP2A/STRA6/CER1/WNT9B/DACT2/EMX2/HAS2/IRX3/GCNT1/MMP17/  LGR4 |
| cognition | GO:0050890 | 9.02E-06 | 0.001213 | 20 | BP | MAPK8IP2/ITGA8/KCNK2/JPH4/PTPRZ1/DKK1/GLP1R/SLC8A2/LRRN4/  SERPINF1/MEIS2/STRA6/DRD2/KIT/SHROOM4/ADCY1/CNTNAP2/  NRXN1/CNTN2/RELN |
| auditory behavior | GO:0031223 | 9.10E-06 | 0.001213 | 5 | BP | STRA6/DRD2/CNTNAP2/NRXN1/SLITRK6 |
| muscle organ development | GO:0007517 | 1.03E-05 | 0.001333 | 24 | BP | FOXC1/CDON/PITX1/TP73/KCNK2/DKK1/HAND1/ID3/EGR1/PAX3/PIM1/  STRA6/ACTA1/MEIS1/EPHB1/FOXH1/ATF3/EOMES/PITX2/ADGRB1/  TBX1/MAFF/DNER/DMD |
| positive regulation of nervous system development | GO:0051962 | 1.14E-05 | 0.001424 | 28 | BP | CNTN1/NGFR/CDON/SEMA3A/RARB/TP73/RAB21/LHX5/CXCL12/DKK1/  SEMA5A/OBSL1/MYRF/FLRT3/SERPINF1/ATP8A2/CRABP2/TPBG/DRD2/  EPHB1/KIT/LRRTM1/ISLR2/IRX3/NRXN1/ADGRB1/SLITRK6/RELN |
| response to mechanical stimulus | GO:0009612 | 1.29E-05 | 0.00156 | 17 | BP | TNC/KCNK2/HPN/CXCL12/ATP8A2/MEIS2/STRA6/ACTA1/DRD2/KIT/  ITGA2/MYD88/CNTNAP2/NRXN1/SLC8A1/SLITRK6/DMD |
| reproductive structure development | GO:0048608 | 1.53E-05 | 0.001796 | 26 | BP | TNC/FOXC1/PRDM1/DLX3/TP63/SEMA3A/GRHL2/COL9A3/HAND1/  SPP1/RDH10/SERPINF1/TBX3/HS6ST1/CTSV/STRA6/LHX9/KIT/WNT9B/  EOMES/PITX2/HTRA1/RARG/IRX5/ZFP42/LGR4 |
| reproductive system development | GO:0061458 | 1.73E-05 | 0.00197 | 26 | BP | TNC/FOXC1/PRDM1/DLX3/TP63/SEMA3A/GRHL2/COL9A3/HAND1/  SPP1/RDH10/SERPINF1/TBX3/HS6ST1/CTSV/STRA6/LHX9/KIT/WNT9B/  EOMES/PITX2/HTRA1/RARG/IRX5/ZFP42/LGR4 |
| inner ear morphogenesis | GO:0042472 | 1.92E-05 | 0.002123 | 11 | BP | DLX6/ITGA8/HPN/DLX5/PRRX1/ATP8A2/TFAP2A/MYO7A/TBX1/ALDH1A3/  SLITRK6 |
| kidney development | GO:0001822 | 2.02E-05 | 0.002128 | 19 | BP | FOXC1/RARB/ITGA8/TP73/ID3/EGR1/RDH10/PODXL/SERPINF1/  TFAP2A/STRA6/CER1/WNT9B/DACT2/HAS2/IRX3/GCNT1/MMP17/LGR4 |
| mechanosensory behavior | GO:0007638 | 2.04E-05 | 0.002128 | 5 | BP | STRA6/DRD2/CNTNAP2/NRXN1/SLITRK6 |
| regulation of epidermis development | GO:0045682 | 2.09E-05 | 0.002128 | 10 | BP | FOXC1/CDH3/TP63/GRHL2/FERMT1/GRHL1/MYCN/CTSV/KDF1/MAFF |
| mesenchyme development | GO:0060485 | 2.35E-05 | 0.002306 | 18 | BP | SEMA3F/FOXC1/SEMA3A/GSK3B/HPN/GCNT2/HAND1/RDH10/DPPA4/  ACTA1/CER1/HMGA2/FOXH1/EOMES/PITX2/HAS2/BNC2/TBX1 |
| response to auditory stimulus | GO:0010996 | 2.39E-05 | 0.002306 | 6 | BP | ATP8A2/STRA6/DRD2/CNTNAP2/NRXN1/SLITRK6 |
| neuron death | GO:0070997 | 2.60E-05 | 0.002386 | 21 | BP | BID/NGFR/TP63/TP73/GSK3B/NLRP1/GRIK5/DKK1/TRIM2/EGR1/CLU/  GRM4/SERPINF1/NES/VSTM2L/TFAP2A/SIGMAR1/EPHB1/KCNB1/  TERT/SLC30A10 |
| embryonic camera-type eye development | GO:0031076 | 2.62E-05 | 0.002386 | 7 | BP | RDH10/NES/TFAP2A/STRA6/PITX2/RARG/ALDH1A3 |
| respiratory system development | GO:0060541 | 2.66E-05 | 0.002386 | 16 | BP | TNC/GRHL2/ADAMTS2/ESRP2/CRISPLD2/DLX5/RDH10/DPPA4/MYCN/  HS6ST1/STRA6/PKDCC/PITX2/RARG/ALDH1A3/STK40 |
| learning | GO:0007612 | 2.78E-05 | 0.002441 | 13 | BP | MAPK8IP2/JPH4/DKK1/SLC8A2/LRRN4/MEIS2/STRA6/DRD2/KIT/CNTNAP2/  NRXN1/CNTN2/RELN |
| odontogenesis of dentin-containing tooth | GO:0042475 | 3.60E-05 | 0.003026 | 10 | BP | TNC/FOXC1/DLX3/TP63/FAM20A/HAND1/PITX2/HTRA1/SOSTDC1/TBX1 |
| odontogenesis | GO:0042476 | 3.60E-05 | 0.003026 | 12 | BP | TNC/FOXC1/DLX3/TP63/FAM20A/HAND1/ID3/TFAP2A/PITX2/HTRA1/  SOSTDC1/TBX1 |
| ear development | GO:0043583 | 4.58E-05 | 0.003762 | 16 | BP | DLX6/ITGA8/KCNK2/HPN/DLX5/PRRX1/RDH10/ATP8A2/MYCN/TFAP2A/  MYO7A/STRA6/CXCL14/TBX1/ALDH1A3/SLITRK6 |
| mesoderm morphogenesis | GO:0048332 | 5.35E-05 | 0.004309 | 9 | BP | FOXC1/EYA2/ITGA8/DKK1/HAND1/TBX3/HMGA2/EOMES/ITGA2 |
| embryonic hindlimb morphogenesis | GO:0035116 | 5.90E-05 | 0.00465 | 6 | BP | PITX1/TP63/RARB/TBX3/PITX2/RARG |
| cardiac muscle tissue development | GO:0048738 | 6.15E-05 | 0.004756 | 15 | BP | FOXC1/RARB/NEBL/TP73/KCNK2/DKK1/HAND1/OBSL1/TBX3/PIM1/  MEIS1/FOXH1/PITX2/NKX2-6/SLC8A1 |
| digestive tract development | GO:0048565 | 6.31E-05 | 0.004779 | 12 | BP | CPS1/PRDM1/TP63/RARB/RARRES2/STRA6/PDGFC/KIT/PKDCC/  PITX2/NKX2-6/LGR4 |
| olfactory bulb development | GO:0021772 | 7.22E-05 | 0.005366 | 6 | BP | SEMA3A/DLX5/NR2E1/EOMES/ATF5/CSF1R |
| regulation of epidermal cell differentiation | GO:0045604 | 7.56E-05 | 0.005512 | 8 | BP | FOXC1/TP63/GRHL2/GRHL1/MYCN/CTSV/KDF1/MAFF |
| negative regulation of neuron differentiation | GO:0045665 | 7.81E-05 | 0.005589 | 15 | BP | SEMA3F/FSTL4/NGFR/SEMA3A/TP73/GSK3B/NR2E1/SEMA5A/SPP1/  SOX3/MEIS1/CNTN4/IRX3/CNTN2/RTN4RL1 |
| embryonic cranial skeleton morphogenesis | GO:0048701 | 8.42E-05 | 0.005919 | 7 | BP | GRHL2/PRRX1/RDH10/TFAP2A/WNT9B/IRX5/TBX1 |
| olfactory lobe development | GO:0021988 | 8.77E-05 | 0.006053 | 6 | BP | SEMA3A/DLX5/NR2E1/EOMES/ATF5/CSF1R |
| glutamate receptor signaling pathway | GO:0007215 | 0.000102 | 0.006839 | 9 | BP | MAPK8IP2/NECAB2/GRIN2D/GRIK5/GRM4/CACNG8/KCNB1/GRIK3/RELN |
| ear morphogenesis | GO:0042471 | 0.000105 | 0.006839 | 11 | BP | DLX6/ITGA8/HPN/DLX5/PRRX1/ATP8A2/TFAP2A/MYO7A/TBX1/  ALDH1A3/SLITRK6 |
| embryonic forelimb morphogenesis | GO:0035115 | 0.000106 | 0.006839 | 6 | BP | TP63/RDH10/TBX3/TFAP2A/CRABP2/WNT9A |
| gland development | GO:0048732 | 0.000106 | 0.006839 | 24 | BP | CPS1/TNC/FOXC1/PKM/PITX1/TP63/SEMA3A/ESRP2/HPN/SERPINF1/  SOX3/TBX3/STRA6/RPL32/DRD2/PITX2/ITGA2/STAT6/SOSTDC1/RARG/  UCP2/CSF1R/TBX1/ALDH1A3 |
| regulation of stem cell proliferation | GO:0072091 | 0.000109 | 0.006905 | 8 | BP | FBLN1/FERMT1/NR2E1/TBX3/DRD2/HMGA2/TERT/KDF1 |
| muscle cell differentiation | GO:0042692 | 0.000112 | 0.007003 | 22 | BP | CDON/PITX1/RARB/ITGA8/NEBL/DKK1/ID3/OBSL1/FLNC/TBX3/TMOD3/  ACTA1/CXCL14/KIT/PITX2/SOSTDC1/NKX2-6/ADGRB1/SLC8A1/TBX1/  DNER/DMD |
| negative regulation of neurogenesis | GO:0050768 | 0.000114 | 0.007021 | 17 | BP | SEMA3F/FSTL4/NGFR/SEMA3A/TP73/GSK3B/NR2E1/SEMA5A/SPP1/  MYCN/SOX3/MEIS1/CNTN4/TERT/IRX3/CNTN2/RTN4RL1 |
| inner ear development | GO:0048839 | 0.000141 | 0.00854 | 14 | BP | DLX6/ITGA8/KCNK2/HPN/DLX5/PRRX1/ATP8A2/MYCN/TFAP2A/MYO7A/  CXCL14/TBX1/ALDH1A3/SLITRK6 |
| embryonic eye morphogenesis | GO:0048048 | 0.000151 | 0.008612 | 6 | BP | RARB/EFEMP1/TFAP2A/STRA6/RARG/ALDH1A3 |
| regulation of neuron death | GO:1901214 | 0.000151 | 0.008612 | 18 | BP | TP63/TP73/GSK3B/GRIK5/DKK1/TRIM2/EGR1/CLU/GRM4/SERPINF1/  NES/VSTM2L/TFAP2A/SIGMAR1/EPHB1/KCNB1/TERT/SLC30A10 |
| axon guidance | GO:0007411 | 0.000152 | 0.008612 | 16 | BP | SEMA3F/NGFR/SEMA3A/DLX5/CXCL12/SEMA5A/DOK4/FLRT3/LHX9/  CNTN4/EPHB1/DPYSL5/NRXN1/CSF1R/CNTN2/RELN |
| forebrain generation of neurons | GO:0021872 | 0.000154 | 0.008612 | 8 | BP | SEMA3A/LHX5/DLX5/NR2E1/DRD2/ATF5/CSF1R/CNTN2 |
| cranial skeletal system development | GO:1904888 | 0.000154 | 0.008612 | 8 | BP | TP63/GRHL2/PRRX1/RDH10/TFAP2A/WNT9B/IRX5/TBX1 |
| digestive system development | GO:0055123 | 0.000161 | 0.008895 | 12 | BP | CPS1/PRDM1/TP63/RARB/RARRES2/STRA6/PDGFC/KIT/PKDCC/  PITX2/NKX2-6/LGR4 |
| neuron projection guidance | GO:0097485 | 0.000168 | 0.00912 | 16 | BP | SEMA3F/NGFR/SEMA3A/DLX5/CXCL12/SEMA5A/DOK4/FLRT3/LHX9/  CNTN4/EPHB1/DPYSL5/NRXN1/CSF1R/CNTN2/RELN |
| regulation of cell morphogenesis involved in differentiation | GO:0010769 | 0.000176 | 0.009393 | 17 | BP | SEMA3F/FSTL4/NGFR/SEMA3A/FBLN1/RAB21/CXCL12/NR2E1/SEMA5A/  SPP1/OBSL1/CRABP2/ISLR2/HAS2/SSH3/CNTN2/RELN |
| skeletal muscle organ development | GO:0060538 | 0.000179 | 0.009393 | 13 | BP | CDON/PITX1/DKK1/EGR1/STRA6/ACTA1/EPHB1/ATF3/EOMES/PITX2/  TBX1/MAFF/DNER |
| skeletal system morphogenesis | GO:0048705 | 0.00018 | 0.009393 | 15 | BP | FOXC1/RARB/GRHL2/DLX5/PRRX1/RDH10/MYCN/TFAP2A/WNT9A/  CER1/WNT9B/HAS2/RARG/IRX5/TBX1 |
| negative regulation of cell development | GO:0010721 | 0.000197 | 0.010155 | 18 | BP | SEMA3F/FSTL4/NGFR/SEMA3A/FBLN1/TP73/GSK3B/NR2E1/SEMA5A/  SPP1/MYCN/SOX3/MEIS1/CNTN4/TERT/IRX3/CNTN2/RTN4RL1 |
| negative chemotaxis | GO:0050919 | 0.000209 | 0.010647 | 6 | BP | SEMA3F/SEMA3A/SEMA5A/FLRT3/SEMA3D/SEMA3E |
| Wnt signaling pathway | GO:0016055 | 0.000218 | 0.010924 | 25 | BP | CDH3/TLE2/GSK3B/FERMT1/AES/DLX5/DKK1/RNF43/EGR1/LGR6/  ARHGEF19/WNT9A/DRD2/LRRK1/WNT9B/PITX2/TERT/DACT2/RSPO1/  SOX7/SOSTDC1/MLLT3/RARG/SHISA2/LGR4 |
| cell-cell signaling by wnt | GO:0198738 | 0.000232 | 0.011491 | 25 | BP | CDH3/TLE2/GSK3B/FERMT1/AES/DLX5/DKK1/RNF43/EGR1/LGR6/  ARHGEF19/WNT9A/DRD2/LRRK1/WNT9B/PITX2/TERT/DACT2/RSPO1/  SOX7/SOSTDC1/MLLT3/RARG/SHISA2/LGR4 |
| morphogenesis of a branching structure | GO:0001763 | 0.000235 | 0.011491 | 14 | BP | TNC/PRDM1/TP63/SEMA3A/GRHL2/ESRP2/RDH10/MYCN/TBX3/DRD2/  WNT9B/PITX2/SEMA3E/LGR4 |
| hindlimb morphogenesis | GO:0035137 | 0.000245 | 0.011828 | 6 | BP | PITX1/TP63/RARB/TBX3/PITX2/RARG |
| mesoderm formation | GO:0001707 | 0.000261 | 0.012469 | 8 | BP | FOXC1/EYA2/ITGA8/DKK1/HAND1/HMGA2/EOMES/ITGA2 |
| regulation of receptor internalization | GO:0002090 | 0.000285 | 0.013231 | 6 | BP | NECAB2/DKK1/DRD2/LRRTM1/SYK/RSPO1 |
| lung development | GO:0030324 | 0.000288 | 0.013231 | 13 | BP | TNC/GRHL2/ADAMTS2/ESRP2/CRISPLD2/RDH10/DPPA4/MYCN/  HS6ST1/STRA6/PKDCC/PITX2/STK40 |
| negative regulation of nervous system development | GO:0051961 | 0.00029 | 0.013231 | 17 | BP | SEMA3F/FSTL4/NGFR/SEMA3A/TP73/GSK3B/NR2E1/SEMA5A/SPP1/  MYCN/SOX3/MEIS1/CNTN4/TERT/IRX3/CNTN2/RTN4RL1 |
| regulation of synapse organization | GO:0050807 | 0.000293 | 0.013231 | 11 | BP | DKK1/FLRT3/LRRC4/TPBG/DRD2/EPHB1/LRRTM1/NRXN1/ADGRB1/  SLITRK6/RELN |
| tube morphogenesis | GO:0035239 | 0.000295 | 0.013231 | 20 | BP | TNC/TP63/GRHL2/ESRP2/HAND1/RDH10/PODXL/MYCN/TBX3/STRA6/  WNT9B/FOXH1/PITX2/SEMA3E/SOSTDC1/RARG/IRX3/CSF1R/TBX1/LGR4 |
| neuron apoptotic process | GO:0051402 | 0.000299 | 0.013231 | 15 | BP | BID/NGFR/TP63/TP73/NLRP1/GRIK5/TRIM2/GRM4/NES/VSTM2L/TFAP2A/  SIGMAR1/KCNB1/TERT/SLC30A10 |
| camera-type eye morphogenesis | GO:0048593 | 0.000301 | 0.013231 | 10 | BP | CDON/ATP8A2/TFAP2A/STRA6/WNT9A/MEIS1/EPHB1/PITX2/IRX5/  ALDH1A3 |
| regulation of extent of cell growth | GO:0061387 | 0.000311 | 0.013274 | 9 | BP | SEMA3F/FSTL4/SEMA3A/RAB21/CXCL12/SEMA5A/SPP1/CRABP2/  ISLR2 |
| biomineral tissue development | GO:0031214 | 0.000314 | 0.013274 | 11 | BP | FAM20A/SPP1/TFAP2A/CER1/PTH1R/PKDCC/NELL1/SLC8A1/GAS6/  TBX1/LGR4 |
| canonical Wnt signaling pathway | GO:0060070 | 0.000314 | 0.013274 | 18 | BP | CDH3/TLE2/GSK3B/FERMT1/AES/DLX5/DKK1/EGR1/LGR6/WNT9A/  LRRK1/WNT9B/RSPO1/SOX7/SOSTDC1/MLLT3/RARG/LGR4 |
| forebrain neuron differentiation | GO:0021879 | 0.000316 | 0.013274 | 7 | BP | SEMA3A/LHX5/DLX5/DRD2/ATF5/CSF1R/CNTN2 |
| regulation of epithelial cell differentiation | GO:0030856 | 0.000335 | 0.013935 | 11 | BP | FOXC1/TP63/GSK3B/GRHL2/GRHL1/MYCN/TBX3/CTSV/WNT9B/KDF1/  MAFF |
| regulation of axonogenesis | GO:0050770 | 0.000346 | 0.014241 | 12 | BP | SEMA3F/FSTL4/NGFR/SEMA3A/RAB21/CXCL12/SEMA5A/SPP1/  CRABP2/ISLR2/SSH3/CNTN2 |
| respiratory tube development | GO:0030323 | 0.00036 | 0.014641 | 13 | BP | TNC/GRHL2/ADAMTS2/ESRP2/CRISPLD2/RDH10/DPPA4/MYCN/  HS6ST1/STRA6/PKDCC/PITX2/STK40 |
| embryonic skeletal system morphogenesis | GO:0048704 | 0.000368 | 0.014798 | 9 | BP | GRHL2/PRRX1/RDH10/MYCN/TFAP2A/WNT9A/WNT9B/IRX5/TBX1 |
| striated muscle cell differentiation | GO:0051146 | 0.000375 | 0.014921 | 16 | BP | CDON/RARB/NEBL/DKK1/OBSL1/FLNC/TBX3/TMOD3/ACTA1/PITX2/  NKX2-6/ADGRB1/SLC8A1/TBX1/DNER/DMD |
| forelimb morphogenesis | GO:0035136 | 0.00038 | 0.014991 | 6 | BP | TP63/RDH10/TBX3/TFAP2A/CRABP2/WNT9A |
| forebrain cell migration | GO:0021885 | 0.000397 | 0.015384 | 7 | BP | SEMA3A/CXCL12/NR2E1/DRD2/EMX2/CNTN2/RELN |
| palate development | GO:0060021 | 0.000399 | 0.015384 | 9 | BP | DLX6/DLX5/PRRX1/TBX3/TFAP2A/WNT9B/PKDCC/BNC2/TBX1 |
| stem cell proliferation | GO:0072089 | 0.000404 | 0.015384 | 10 | BP | FBLN1/FERMT1/RNF43/NR2E1/NES/TBX3/DRD2/HMGA2/TERT/KDF1 |
| regulation of synapse structure or activity | GO:0050803 | 0.000408 | 0.015384 | 11 | BP | DKK1/FLRT3/LRRC4/TPBG/DRD2/EPHB1/LRRTM1/NRXN1/ADGRB1/  SLITRK6/RELN |
| skeletal muscle tissue development | GO:0007519 | 0.000414 | 0.015384 | 12 | BP | CDON/PITX1/DKK1/EGR1/ACTA1/EPHB1/ATF3/EOMES/PITX2/TBX1/  MAFF/DNER |
| regulation of synaptic plasticity | GO:0048167 | 0.000414 | 0.015384 | 12 | BP | SYT7/JPH4/NR2E1/SLC8A2/CNTN4/DRD2/KIT/KCNB1/LRRTM1/  ADGRB1/CNTN2/RELN |
| cartilage development | GO:0051216 | 0.000424 | 0.015599 | 13 | BP | PITX1/RARB/HAND1/EFEMP1/PRRX1/TGFBI/MYCN/WNT9A/CER1/  HMGA2/PTH1R/PKDCC/RARG |
| positive regulation of cell motility | GO:2000147 | 0.000461 | 0.016818 | 23 | BP | CD74/SEMA3A/RARRES2/CXCL12/GCNT2/SEMA5A/PLAU/PODXL/  RAB25/LGR6/SEMA6D/PDGFC/CXCL14/KIT/CARMIL2/ITGA2/TERT/  HAS2/CAVIN1/CSF1R/SLC8A1/GAS6/RELN |
| striatum development | GO:0021756 | 0.000498 | 0.017481 | 4 | BP | RARB/DRD2/CNTNAP2/ALDH1A3 |
| preganglionic parasympathetic fiber development | GO:0021783 | 0.000498 | 0.017481 | 4 | BP | SEMA3F/SEMA3A/TFAP2A/TBX1 |
| nose development | GO:0043584 | 0.000498 | 0.017481 | 4 | BP | DLX5/RDH10/STRA6/ALDH1A3 |
| regulation of axon extension involved in axon guidance | GO:0048841 | 0.000498 | 0.017481 | 4 | BP | SEMA3F/SEMA3A/CXCL12/SEMA5A |
| diencephalon development | GO:0021536 | 0.000509 | 0.017704 | 8 | BP | PITX1/SEMA3A/SEMA5A/SOX3/DRD2/PITX2/CNTNAP2/NKX2-6 |
| cranial nerve morphogenesis | GO:0021602 | 0.000514 | 0.017746 | 5 | BP | SEMA3F/SEMA3A/TFAP2A/EPHB1/TBX1 |
| regulation of system process | GO:0044057 | 0.000532 | 0.018178 | 25 | BP | ACPP/ATP2B3/ATP2B1/SEMA3A/KCNK6/TNNT1/GRIN2D/GLP1R/  SLC8A2/MYRF/CACNG8/DRD2/TWF1/RAB11FIP1/CACNA1D/KIT/  GUCY1A3/ITGA2/SLN/IRX5/SLC8A1/GAS6/KCND2/CCK/DMD |
| neuromuscular process | GO:0050905 | 0.000547 | 0.018198 | 9 | BP | JPH4/GRIN2D/ATP8A2/MYO7A/STRA6/DRD2/NRXN1/ALDH1A3/  SLITRK6 |
| regulation of synaptic transmission, glutamatergic | GO:0051966 | 0.000549 | 0.018198 | 7 | BP | MAPK8IP2/DKK1/GRM4/DRD2/GRIK3/NRXN1/RELN |
| bone development | GO:0060348 | 0.000552 | 0.018198 | 13 | BP | FOXC1/RARB/DLX5/TFAP2A/MEIS1/PDGFC/CER1/LRRK1/KIT/PITX2/  HAS2/RARG/SPNS2 |
| synaptic transmission, glutamatergic | GO:0035249 | 0.000556 | 0.018198 | 8 | BP | MAPK8IP2/GRIK5/DKK1/GRM4/DRD2/GRIK3/NRXN1/RELN |
| negative regulation of developmental growth | GO:0048640 | 0.000556 | 0.018198 | 8 | BP | SEMA3F/FSTL4/SEMA3A/TP73/KCNK2/SEMA5A/SPP1/MEIS1 |
| modulation of chemical synaptic transmission | GO:0050804 | 0.000569 | 0.018491 | 18 | BP | MAPK8IP2/SYT7/JPH4/GRIK5/DKK1/NR2E1/SLC8A2/GRM4/CNTN4/  DRD2/KIT/KCNB1/LRRTM1/GRIK3/NRXN1/ADGRB1/CNTN2/RELN |
| synapse organization | GO:0050808 | 0.000603 | 0.019432 | 16 | BP | TNC/DKK1/FLRT3/LRRC4/TPBG/DRD2/EPHB1/LRRTM1/SEMA3E/  NRXN1/ADGRB1/CNTN2/SLITRK6/DNER/COL4A5/RELN |
| formation of primary germ layer | GO:0001704 | 0.000612 | 0.019544 | 10 | BP | FOXC1/EYA2/ITGA8/DKK1/HAND1/HMGA2/EOMES/ITGA2/SOX7/  POU5F1 |
| positive regulation of cell migration | GO:0030335 | 0.00063 | 0.019576 | 22 | BP | CD74/SEMA3A/RARRES2/CXCL12/GCNT2/SEMA5A/PLAU/PODXL/  RAB25/LGR6/SEMA6D/PDGFC/CXCL14/KIT/CARMIL2/ITGA2/TERT/  HAS2/CSF1R/SLC8A1/GAS6/RELN |
| striated muscle cell development | GO:0055002 | 0.000631 | 0.019576 | 11 | BP | NEBL/OBSL1/FLNC/TBX3/TMOD3/ACTA1/PITX2/NKX2-6/SLC8A1/  DNER/DMD |
| central nervous system myelination | GO:0022010 | 0.000638 | 0.019576 | 4 | BP | CLU/PLP1/MYRF/CNTN2 |
| axon ensheathment in central nervous system | GO:0032291 | 0.000638 | 0.019576 | 4 | BP | CLU/PLP1/MYRF/CNTN2 |
| negative regulation of epidermis development | GO:0045683 | 0.000638 | 0.019576 | 4 | BP | CDH3/TP63/GRHL2/FERMT1 |
| positive regulation of cellular component movement | GO:0051272 | 0.00065 | 0.01959 | 23 | BP | CD74/SEMA3A/RARRES2/CXCL12/GCNT2/SEMA5A/PLAU/PODXL/  RAB25/LGR6/SEMA6D/PDGFC/CXCL14/KIT/CARMIL2/ITGA2/TERT/  HAS2/CAVIN1/CSF1R/SLC8A1/GAS6/RELN |
| positive regulation of epithelial cell proliferation | GO:0050679 | 0.000651 | 0.01959 | 12 | BP | DLX6/CDH3/TP63/ESRP2/HPN/DLX5/CXCL12/SEMA5A/HTRA1/HAS2/  EGFL7/TBX1 |
| gland morphogenesis | GO:0022612 | 0.000654 | 0.01959 | 10 | BP | TNC/TP63/SEMA3A/ESRP2/HPN/TBX3/STAT6/SOSTDC1/RARG/CSF1R |
| regulation of receptor-mediated endocytosis | GO:0048259 | 0.000661 | 0.019635 | 8 | BP | RAB21/NECAB2/DKK1/APOC1/DRD2/LRRTM1/SYK/RSPO1 |
| regulation of Wnt signaling pathway | GO:0030111 | 0.000713 | 0.02103 | 18 | BP | TLE2/GSK3B/FERMT1/AES/DLX5/DKK1/RNF43/EGR1/LGR6/LRRK1/  TERT/DACT2/RSPO1/SOX7/SOSTDC1/MLLT3/SHISA2/LGR4 |
| epithelial cell proliferation | GO:0050673 | 0.000747 | 0.021876 | 19 | BP | DLX6/CDH3/NGFR/TP63/FERMT1/ESRP2/HPN/DLX5/CXCL12/SEMA5A/  SERPINF1/KIT/HTRA1/STAT6/HAS2/EGFL7/KDF1/TBX1/LGR4 |
| regulation of cell morphogenesis | GO:0022604 | 0.000783 | 0.022761 | 22 | BP | SEMA3F/FSTL4/NGFR/SEMA3A/FBLN1/RAB21/PALMD/HPN/CXCL12/  NR2E1/SEMA5A/SPP1/OBSL1/CRABP2/KIT/ISLR2/SEMA3E/HAS2/SSH3/  CSF1R/CNTN2/RELN |
| parasympathetic nervous system development | GO:0048486 | 0.000805 | 0.023043 | 4 | BP | SEMA3F/SEMA3A/TFAP2A/TBX1 |
| positive regulation of protein depolymerization | GO:1901881 | 0.000805 | 0.023043 | 4 | BP | SEMA5A/NES/CARMIL2/SLN |
| face morphogenesis | GO:0060325 | 0.000838 | 0.023797 | 5 | BP | CRISPLD2/DLX5/DKK1/STRA6/TBX1 |
| cell fate commitment | GO:0045165 | 0.000929 | 0.026191 | 15 | BP | PRDM1/CDON/EYA2/PITX1/DKK1/NR2E1/PRRX1/TBX3/WNT9A/WNT9B/  EOMES/STAT6/SOSTDC1/TBX1/POU5F1 |
| central nervous system neuron differentiation | GO:0021953 | 0.000943 | 0.026402 | 12 | BP | SEMA3A/LHX5/DLX5/DKK1/NR2E1/DRD2/EPHB1/WNT9B/EOMES/ATF5/  CSF1R/CNTN2 |
| epithelial cell development | GO:0002064 | 0.000952 | 0.026453 | 13 | BP | PRDM1/TP63/FOSL2/RARB/GSK3B/GRHL2/PODXL/RAB25/MYO7A/  DACT2/RARG/KDF1/SLITRK6 |
| neuron recognition | GO:0008038 | 0.000973 | 0.026603 | 5 | BP | SEMA3A/SEMA5A/CNTN4/CNTNAP2/CNTN2 |
| mesodermal cell differentiation | GO:0048333 | 0.000973 | 0.026603 | 5 | BP | EYA2/ITGA8/DKK1/HMGA2/ITGA2 |
| columnar/cuboidal epithelial cell differentiation | GO:0002065 | 0.000978 | 0.026603 | 9 | BP | PRDM1/DLX3/TP63/RARB/GSK3B/MYCN/MYO7A/RARG/SLITRK6 |
| regulation of cell size | GO:0008361 | 0.001 | 0.026842 | 11 | BP | SEMA3F/FSTL4/SEMA3A/RAB21/CXCL12/SEMA5A/SPP1/VAV3/  CRABP2/ISLR2/RARG |
| negative regulation of chondrocyte differentiation | GO:0032331 | 0.001 | 0.026842 | 4 | BP | RARB/EFEMP1/WNT9A/RARG |
| skeletal muscle cell differentiation | GO:0035914 | 0.001083 | 0.028662 | 7 | BP | CDON/EGR1/EPHB1/ATF3/EOMES/TBX1/MAFF |
| positive regulation of synapse assembly | GO:0051965 | 0.001083 | 0.028662 | 7 | BP | FLRT3/TPBG/EPHB1/LRRTM1/NRXN1/ADGRB1/SLITRK6 |
| positive regulation of locomotion | GO:0040017 | 0.001134 | 0.029807 | 23 | BP | CD74/SEMA3A/RARRES2/CXCL12/GCNT2/SEMA5A/PLAU/PODXL/  RAB25/LGR6/SEMA6D/PDGFC/CXCL14/KIT/CARMIL2/ITGA2/TERT/  HAS2/CAVIN1/CSF1R/SLC8A1/GAS6/RELN |
| cranial nerve development | GO:0021545 | 0.001153 | 0.030092 | 6 | BP | SEMA3F/SEMA3A/TFAP2A/EPHB1/TBX1/SLITRK6 |
| heart development | GO:0007507 | 0.001166 | 0.030239 | 24 | BP | FOXC1/PRDM1/RARB/NEBL/TP73/KCNK2/GRHL2/DKK1/HAND1/ID3/  OBSL1/FLRT3/TBX3/PIM1/STRA6/MEIS1/CER1/FOXH1/EOMES/PITX2/  HAS2/NKX2-6/SLC8A1/TBX1 |
| regionalization | GO:0003002 | 0.001214 | 0.031181 | 18 | BP | SEMA3F/FOXC1/CDON/TP63/SEMA3A/DKK1/TBX3/CER1/FOXH1/  EOMES/PITX2/EMX2/SOSTDC1/MLLT3/RARG/IRX3/TBX1/RELN |
| axonal fasciculation | GO:0007413 | 0.001227 | 0.031181 | 4 | BP | SEMA3A/SEMA5A/CNTN4/CNTN2 |
| neuron projection fasciculation | GO:0106030 | 0.001227 | 0.031181 | 4 | BP | SEMA3A/SEMA5A/CNTN4/CNTN2 |
| stem cell population maintenance | GO:0019827 | 0.001243 | 0.031399 | 11 | BP | TP63/NR2E1/PRRX1/DPPA4/ZSCAN10/TBX3/HMGA2/KIT/WNT9B/  EOMES/POU5F1 |
| cerebral cortex development | GO:0021987 | 0.001279 | 0.031801 | 9 | BP | CDON/SEMA3A/GSK3B/NR2E1/EOMES/EMX2/GNG12/CNTNAP2/  RELN |
| body morphogenesis | GO:0010171 | 0.001283 | 0.031801 | 6 | BP | CDON/CRISPLD2/DLX5/DKK1/STRA6/TBX1 |
| positive regulation of protein complex disassembly | GO:0043243 | 0.001292 | 0.031801 | 5 | BP | CLEC16A/SEMA5A/NES/CARMIL2/SLN |
| embryonic digestive tract development | GO:0048566 | 0.001292 | 0.031801 | 5 | BP | RARB/RARRES2/STRA6/PKDCC/PITX2 |
| cardiac chamber development | GO:0003205 | 0.001311 | 0.032064 | 11 | BP | FOXC1/PRDM1/RARB/KCNK2/GRHL2/HAND1/TBX3/STRA6/FOXH1/  PITX2/TBX1 |
| morphogenesis of a branching epithelium | GO:0061138 | 0.001337 | 0.032482 | 12 | BP | TNC/TP63/SEMA3A/GRHL2/ESRP2/RDH10/MYCN/TBX3/WNT9B/  PITX2/SEMA3E/LGR4 |
| developmental growth involved in morphogenesis | GO:0060560 | 0.001375 | 0.033166 | 13 | BP | SEMA3F/TNC/FSTL4/SEMA3A/RAB21/CXCL12/SEMA5A/SPP1/  RDH10/FLRT3/CRABP2/ISLR2/RARG |
| maintenance of cell number | GO:0098727 | 0.001382 | 0.033166 | 11 | BP | TP63/NR2E1/PRRX1/DPPA4/ZSCAN10/TBX3/HMGA2/KIT/WNT9B/  EOMES/POU5F1 |
| epithelial tube morphogenesis | GO:0060562 | 0.001396 | 0.033307 | 17 | BP | TNC/GRHL2/ESRP2/HAND1/RDH10/PODXL/MYCN/TBX3/WNT9B/  FOXH1/PITX2/SEMA3E/SOSTDC1/RARG/IRX3/CSF1R/LGR4 |
| regulation of anatomical structure size | GO:0090066 | 0.001415 | 0.033324 | 22 | BP | SEMA3F/CPS1/FSTL4/FOXC1/SEMA3A/RAB21/CXCL12/SEMA5A/  SPP1/NTS/VAV3/TMOD3/CRABP2/TWF1/WNT9B/CARMIL2/GUCY1A3/  ISLR2/RARG/SSH3/SLC8A1/CNTN2 |
| negative regulation of axonogenesis | GO:0050771 | 0.001425 | 0.033324 | 6 | BP | SEMA3F/FSTL4/NGFR/SEMA3A/SEMA5A/SPP1 |
| lung morphogenesis | GO:0060425 | 0.001425 | 0.033324 | 6 | BP | TNC/GRHL2/ESRP2/RDH10/PITX2/STK40 |
| muscle cell development | GO:0055001 | 0.001456 | 0.033324 | 11 | BP | NEBL/OBSL1/FLNC/TBX3/TMOD3/ACTA1/PITX2/NKX2-6/SLC8A1/  DNER/DMD |
| hypothalamus development | GO:0021854 | 0.001486 | 0.033324 | 4 | BP | SEMA3A/SOX3/PITX2/NKX2-6 |
| cerebral cortex neuron differentiation | GO:0021895 | 0.001486 | 0.033324 | 4 | BP | NR2E1/DRD2/EOMES/CNTN2 |
| tongue development | GO:0043586 | 0.001486 | 0.033324 | 4 | BP | KIT/BNC2/NKX2-6/TBX1 |
| axon extension involved in axon guidance | GO:0048846 | 0.001486 | 0.033324 | 4 | BP | SEMA3F/SEMA3A/CXCL12/SEMA5A |
| neuron projection extension involved in neuron projection guidance | GO:1902284 | 0.001486 | 0.033324 | 4 | BP | SEMA3F/SEMA3A/CXCL12/SEMA5A |
| regulation of axon guidance | GO:1902667 | 0.001486 | 0.033324 | 4 | BP | SEMA3F/SEMA3A/CXCL12/SEMA5A |
| diterpenoid biosynthetic process | GO:0016102 | 0.001509 | 0.033324 | 3 | BP | RDH10/CRABP2/ALDH1A3 |
| learned vocalization behavior or vocal learning | GO:0098598 | 0.001509 | 0.033324 | 3 | BP | STRA6/CNTNAP2/NRXN1 |
| negative regulation of axon guidance | GO:1902668 | 0.001509 | 0.033324 | 3 | BP | SEMA3F/SEMA3A/SEMA5A |
| somatic stem cell population maintenance | GO:0035019 | 0.001534 | 0.033688 | 7 | BP | TP63/NR2E1/DPPA4/ZSCAN10/KIT/WNT9B/POU5F1 |
| columnar/cuboidal epithelial cell development | GO:0002066 | 0.001579 | 0.03446 | 6 | BP | PRDM1/RARB/GSK3B/MYO7A/RARG/SLITRK6 |
| negative regulation of chemotaxis | GO:0050922 | 0.001682 | 0.036521 | 5 | BP | SEMA3F/SEMA3A/C5/SEMA5A/PADI2 |
| regulation of developmental growth | GO:0048638 | 0.001717 | 0.036938 | 16 | BP | SEMA3F/FSTL4/FOXC1/SEMA3A/TP73/RAB21/KCNK2/CXCL12/  SEMA5A/SPP1/ATP8A2/PIM1/CRABP2/MEIS1/DRD2/ISLR2 |
| response to extracellular stimulus | GO:0009991 | 0.001721 | 0.036938 | 22 | BP | CPS1/CLEC16A/TNC/PKM/ATP2B1/AES/SPP1/LDHA/CTSV/PIM1/  ACTA1/KCNB1/WNT9B/ATF3/NUAK2/PITX2/ITGA2/FOXA3/UCP2/  SLC8A1/GAS6/CCK |
| telencephalon cell migration | GO:0022029 | 0.001744 | 0.037067 | 6 | BP | SEMA3A/CXCL12/NR2E1/DRD2/CNTN2/RELN |
| connective tissue development | GO:0061448 | 0.001753 | 0.037067 | 14 | BP | PITX1/RARB/HAND1/EFEMP1/PRRX1/TGFBI/EGR1/MYCN/WNT9A/  CER1/HMGA2/PTH1R/PKDCC/RARG |
| female sex differentiation | GO:0046660 | 0.001755 | 0.037067 | 9 | BP | FOXC1/TP63/COL9A3/TBX3/STRA6/LHX9/KIT/PITX2/ZFP42 |
| endochondral bone growth | GO:0003416 | 0.001782 | 0.037223 | 4 | BP | RARB/CER1/RARG/BNC2 |
| subpallium development | GO:0021544 | 0.001782 | 0.037223 | 4 | BP | RARB/DRD2/CNTNAP2/ALDH1A3 |
| mesenchymal cell differentiation | GO:0048762 | 0.001857 | 0.038586 | 12 | BP | SEMA3F/FOXC1/SEMA3A/GSK3B/HPN/GCNT2/RDH10/HMGA2/  EOMES/PITX2/HAS2/TBX1 |
| sensory perception of mechanical stimulus | GO:0050954 | 0.001876 | 0.03876 | 11 | BP | HPN/CXCL12/PAX3/TFAP2A/MYO7A/CACNA1D/KIT/ITGA2/TBX1/  SLITRK6/ESPN |
| keratinocyte proliferation | GO:0043616 | 0.001907 | 0.038898 | 5 | BP | CDH3/TP63/FERMT1/HAS2/KDF1 |
| head morphogenesis | GO:0060323 | 0.001907 | 0.038898 | 5 | BP | CRISPLD2/DLX5/DKK1/STRA6/TBX1 |
| chondrocyte differentiation | GO:0002062 | 0.001922 | 0.038898 | 8 | BP | RARB/EFEMP1/TGFBI/WNT9A/HMGA2/PTH1R/PKDCC/RARG |
| mitochondrial outer membrane permeabilization | GO:0097345 | 0.001923 | 0.038898 | 6 | BP | BID/EYA2/TP63/TP73/GSK3B/HSPA1A |
| cellular response to nutrient levels | GO:0031669 | 0.001944 | 0.039119 | 12 | BP | CLEC16A/TNC/ATP2B1/CTSV/PIM1/KCNB1/WNT9B/ATF3/NUAK2/  FOXA3/UCP2/GAS6 |
| nerve development | GO:0021675 | 0.00196 | 0.039242 | 7 | BP | SEMA3F/SEMA3A/CTSV/TFAP2A/EPHB1/TBX1/SLITRK6 |
| angiogenesis | GO:0001525 | 0.001976 | 0.039362 | 21 | BP | FOXC1/NGFR/C5/NR2E1/SEMA5A/HAND1/TGFBI/SERPINF1/VAV3/  HS6ST1/MEIS1/HMGA2/DDAH1/EPHB1/PITX2/TERT/SYK/SEMA3E/  EGFL7/ADGRB1/TBX1 |
| collagen-activated signaling pathway | GO:0038065 | 0.002038 | 0.040177 | 3 | BP | ITGA2/SYK/COL4A5 |
| negative regulation of stem cell proliferation | GO:2000647 | 0.002038 | 0.040177 | 3 | BP | FBLN1/FERMT1/KDF1 |
| cardiac ventricle development | GO:0003231 | 0.002104 | 0.041265 | 9 | BP | FOXC1/PRDM1/KCNK2/GRHL2/HAND1/TBX3/STRA6/FOXH1/PITX2 |
| retinoic acid metabolic process | GO:0042573 | 0.002116 | 0.041301 | 4 | BP | RDH10/CRABP2/CRABP1/ALDH1A3 |
| positive regulation of stem cell proliferation | GO:2000648 | 0.002153 | 0.0418 | 5 | BP | NR2E1/TBX3/DRD2/HMGA2/TERT |
| sex differentiation | GO:0007548 | 0.002187 | 0.042245 | 15 | BP | FOXC1/TP63/SEMA3A/COL9A3/RDH10/TBX3/CTSV/STRA6/LHX9/  KIT/WNT9B/PITX2/IRX5/ZFP42/LGR4 |
| diterpenoid metabolic process | GO:0016101 | 0.0022 | 0.042291 | 8 | BP | RARRES2/RDH10/STRA6/CRABP2/ALDH1A1/CRABP1/PDE3A/  ALDH1A3 |
| regulation of canonical Wnt signaling pathway | GO:0060828 | 0.002218 | 0.042315 | 14 | BP | TLE2/GSK3B/FERMT1/AES/DLX5/DKK1/EGR1/LGR6/LRRK1/  RSPO1/SOX7/SOSTDC1/MLLT3/LGR4 |
| response to monosaccharide | GO:0034284 | 0.002223 | 0.042315 | 12 | BP | GRIK5/EGR1/SERPINF1/LDHA/CTSV/COL6A2/KCNB1/ITGA2/  UCP2/NRXN1/SLC8A1/GAS6 |
| cellular response to extracellular stimulus | GO:0031668 | 0.002296 | 0.043484 | 13 | BP | CLEC16A/TNC/ATP2B1/AES/CTSV/PIM1/KCNB1/WNT9B/ATF3/  NUAK2/FOXA3/UCP2/GAS6 |
| positive regulation of mitochondrial membrane permeability involved in apoptotic process | GO:1902110 | 0.002321 | 0.043746 | 6 | BP | BID/EYA2/TP63/TP73/GSK3B/HSPA1A |
| regulation of keratinocyte differentiation | GO:0045616 | 0.002421 | 0.045189 | 5 | BP | FOXC1/TP63/GRHL2/GRHL1/CTSV |
| cellular response to glucagon stimulus | GO:0071377 | 0.002421 | 0.045189 | 5 | BP | CPS1/GLP1R/ADCY1/GNG4/GNG12 |
| negative regulation of myoblast differentiation | GO:0045662 | 0.002491 | 0.045719 | 4 | BP | ID3/TBX3/CXCL14/SOSTDC1 |
| negative regulation of cartilage development | GO:0061037 | 0.002491 | 0.045719 | 4 | BP | RARB/EFEMP1/WNT9A/RARG |
| bone growth | GO:0098868 | 0.002491 | 0.045719 | 4 | BP | RARB/CER1/RARG/BNC2 |
| embryonic skeletal system development | GO:0048706 | 0.002505 | 0.045719 | 9 | BP | GRHL2/PRRX1/RDH10/MYCN/TFAP2A/WNT9A/WNT9B/IRX5/TBX1 |
| cardiac muscle cell differentiation | GO:0055007 | 0.002508 | 0.045719 | 8 | BP | RARB/NEBL/DKK1/OBSL1/TBX3/PITX2/NKX2-6/SLC8A1 |
| adenosine receptor signaling pathway | GO:0001973 | 0.002669 | 0.047299 | 3 | BP | ACPP/NECAB2/CNTN2 |
| terpenoid biosynthetic process | GO:0016114 | 0.002669 | 0.047299 | 3 | BP | RDH10/CRABP2/ALDH1A3 |
| trigeminal nerve development | GO:0021559 | 0.002669 | 0.047299 | 3 | BP | SEMA3F/SEMA3A/TFAP2A |
| negative regulation of cardiac muscle cell proliferation | GO:0060044 | 0.002669 | 0.047299 | 3 | BP | TP73/KCNK2/MEIS1 |
| embryonic camera-type eye formation | GO:0060900 | 0.002669 | 0.047299 | 3 | BP | TFAP2A/STRA6/ALDH1A3 |
| negative regulation of cellular senescence | GO:2000773 | 0.002669 | 0.047299 | 3 | BP | HMGA2/TERT/SLC30A10 |
| positive regulation of cytokine-mediated signaling pathway | GO:0001961 | 0.002712 | 0.047639 | 5 | BP | CD74/AGPAT2/GAS6/HSPA1B/HSPA1A |
| regulation of neurotransmitter receptor activity | GO:0099601 | 0.002712 | 0.047639 | 5 | BP | MAPK8IP2/CACNG8/LYPD6B/LYPD6/RELN |
| regulation of neuron apoptotic process | GO:0043523 | 0.002761 | 0.047922 | 12 | BP | TP63/TP73/GRIK5/TRIM2/GRM4/NES/VSTM2L/TFAP2A/SIGMAR1/  KCNB1/TERT/SLC30A10 |
| striated muscle cell proliferation | GO:0014855 | 0.002778 | 0.047922 | 6 | BP | FOXC1/TP73/KCNK2/PIM1/MEIS1/EPHB1 |
| artery morphogenesis | GO:0048844 | 0.002778 | 0.047922 | 6 | BP | FOXC1/PRDM1/PRRX1/STRA6/FOXH1/TBX1 |
| mitochondrial outer membrane permeabilization involved in programmed cell death | GO:1902686 | 0.002778 | 0.047922 | 6 | BP | BID/EYA2/TP63/TP73/GSK3B/HSPA1A |
| startle response | GO:0001964 | 0.00291 | 0.049749 | 4 | BP | GRIN2D/DRD2/NRXN1/SLITRK6 |
| detection of mechanical stimulus involved in sensory perception | GO:0050974 | 0.00291 | 0.049749 | 4 | BP | HPN/CXCL12/KIT/ITGA2 |
| proteinaceous extracellular matrix | GO:0005578 | 1.50E-07 | 5.61E-05 | 27 | CC | TNC/FBLN1/COL5A3/ADAMTS2/COL9A3/TIMP3/CRISPLD2/  PTPRZ1/EFEMP1/TGFBI/FLRT3/SERPINF1/ADAMTS17/COL6A2/  WNT9A/HAPLN1/SPARCL1/WNT9B/VWA2/EFEMP2/HPSE2/  COL4A5/RELN/COL4A6/MMP17/SMOC1/COL6A6 |
| synaptic membrane | GO:0097060 | 2.35E-05 | 0.003089 | 20 | CC | SYT7/ATP2B1/ITGA8/GRIN2D/GRIK5/GRM4/FLRT3/LRRC4/  CACNG8/SIGMAR1/KCNB1/LRRTM1/GRIK3/KCTD12/NRXN1/  ADGRB1/SLC8A1/KCND2/SH2D5/DMD |
| extracellular matrix component | GO:0044420 | 2.48E-05 | 0.003089 | 12 | CC | TNC/FBLN1/COL5A3/TIMP3/PTPRZ1/TGFBI/SERPINF1/VWA2/  EFEMP2/COL4A5/COL4A6/SMOC1 |
| neuron projection terminus | GO:0044306 | 6.02E-05 | 0.00465 | 12 | CC | SYT7/KCNK2/GRIK5/FLRT3/NTS/MICAL1/DRD2/GRIK3/PRSS12/  ITGA2/CCK/DMD |
| basement membrane | GO:0005604 | 6.65E-05 | 0.00465 | 10 | CC | TNC/FBLN1/TIMP3/TGFBI/SERPINF1/VWA2/EFEMP2/COL4A5/  COL4A6/SMOC1 |
| perikaryon | GO:0043204 | 8.73E-05 | 0.00465 | 11 | CC | ITGA8/GRIK5/SLC8A2/CTSV/DRD2/KCNB1/GRIK3/CNTNAP2/  KCND2/RTN4RL1/CCK |
| axon terminus | GO:0043679 | 8.73E-05 | 0.00465 | 11 | CC | SYT7/KCNK2/GRIK5/FLRT3/NTS/MICAL1/DRD2/GRIK3/PRSS12/  ITGA2/CCK |
| dendrite | GO:0030425 | 0.000127 | 0.005917 | 26 | CC | SYT7/ATP2B1/TP63/SEMA3A/CTTNBP2/ITGA8/JPH4/PALMD/  NECAB2/GRIK5/SLC8A2/CLU/LRRC4/DRD2/EPHB1/DPYSL5/  KCNB1/GRIK3/PRSS12/CNTNAP2/ADGRB1/SLC8A1/KCND2/  CCK/DNER/RELN |
| cell body | GO:0044297 | 0.000149 | 0.006182 | 26 | CC | MAPK8IP2/SYT7/ATP2B1/ITGA8/KCNK2/NLRP1/TUBB4A/HPN/  GRIK5/SLC8A2/RDH10/PODXL/SERPINF1/CTSV/ACTA1/DRD2/  DPYSL5/KCNB1/GRIK3/CNTNAP2/NRXN1/CNTN2/KCND2/RTN4RL1/  CCK/DNER |
| neuronal cell body | GO:0043025 | 0.000285 | 0.009821 | 23 | CC | MAPK8IP2/SYT7/ATP2B1/ITGA8/KCNK2/NLRP1/TUBB4A/HPN/GRIK5/  SLC8A2/SERPINF1/CTSV/DRD2/DPYSL5/KCNB1/GRIK3/CNTNAP2/  NRXN1/CNTN2/KCND2/RTN4RL1/CCK/DNER |
| axon | GO:0030424 | 0.000291 | 0.009821 | 22 | CC | SYT7/SEMA3A/RAB21/KCNK2/NECAB2/TUBB4A/GRIK5/FLRT3/  SERPINF1/MICAL1/CNTN4/DRD2/EPHB1/KCNB1/LRRTM1/GRIK3/  PRSS12/ITGA2/CNTNAP2/IRX3/CNTN2/CCK |
| presynapse | GO:0098793 | 0.000316 | 0.009821 | 20 | CC | DBNDD1/TSPOAP1/SYT7/CTTNBP2/KCNK2/GRIK5/GRM4/FLRT3/  NTS/SYT6/MICAL1/SYTL1/SYTL5/DRD2/GRIK3/PRSS12/ITGA2/  KCTD12/NRXN1/CCK |
| postsynapse | GO:0098794 | 0.000478 | 0.01372 | 22 | CC | MAPK8IP2/ATP2B1/CTTNBP2/ITGA8/GSK3B/PALMD/GRIN2D/  GRIK5/SLC8A2/LRRC4/CACNG8/SIGMAR1/DRD2/KCNB1/LRRTM1/  GRIK3/KCTD12/ADGRB1/SLC8A1/KCND2/SH2D5/DMD |
| cell-substrate junction | GO:0030055 | 0.00062 | 0.016506 | 21 | CC | ARHGAP31/TNC/TLE2/RPL31/ITGA8/RAB21/FERMT1/PLAU/FLRT3/  FLNC/LMO7/TLN1/TWF1/PGM5/CSRP1/ITGA2/ADGRB1/RPS4X/DMD/  HSPA1B/HSPA1A |
| postsynaptic membrane | GO:0045211 | 0.00087 | 0.02164 | 14 | CC | GRIN2D/GRIK5/LRRC4/CACNG8/SIGMAR1/KCNB1/LRRTM1/  GRIK3/KCTD12/ADGRB1/SLC8A1/KCND2/SH2D5/DMD |
| focal adhesion | GO:0005925 | 0.001209 | 0.024396 | 20 | CC | ARHGAP31/TNC/TLE2/RPL31/ITGA8/RAB21/FERMT1/PLAU/FLRT3/  FLNC/LMO7/TLN1/TWF1/PGM5/CSRP1/ITGA2/ADGRB1/RPS4X/  HSPA1B/HSPA1A |
| cell-cell junction | GO:0005911 | 0.001221 | 0.024396 | 21 | CC | CDH3/GRHL2/TJP3/HPN/OBSL1/FLRT3/PODXL/TRPC4/TLN1/  TRIM29/TMOD3/TWF1/PGM5/KIT/S100A11/SMAGP/PLEKHG5/  CNTNAP2/ADGRB1/SLC8A1/PCDH9 |
| axon part | GO:0033267 | 0.001233 | 0.024396 | 12 | CC | SYT7/RAB21/TUBB4A/GRIK5/FLRT3/SERPINF1/MICAL1/GRIK3/  PRSS12/CNTNAP2/CNTN2/CCK |
| dendritic spine | GO:0043197 | 0.001243 | 0.024396 | 10 | CC | ATP2B1/CTTNBP2/ITGA8/PALMD/SLC8A2/LRRC4/DRD2/ADGRB1/  SLC8A1/KCND2 |
| cell-substrate adherens junction | GO:0005924 | 0.001325 | 0.024712 | 20 | CC | ARHGAP31/TNC/TLE2/RPL31/ITGA8/RAB21/FERMT1/PLAU/FLRT3/  FLNC/LMO7/TLN1/TWF1/PGM5/CSRP1/ITGA2/ADGRB1/RPS4X/  HSPA1B/HSPA1A |
| neuron spine | GO:0044309 | 0.001393 | 0.024735 | 10 | CC | ATP2B1/CTTNBP2/ITGA8/PALMD/SLC8A2/LRRC4/DRD2/ADGRB1/  SLC8A1/KCND2 |
| potassium channel complex | GO:0034705 | 0.00235 | 0.039842 | 8 | CC | KCNK2/KCNK6/GRIK5/KCNB1/CNTNAP2/CNTN2/KCND2/KCNQ5 |
| semaphorin receptor binding | GO:0030215 | 1.70E-06 | 0.000973 | 5 | MF | SEMA3A/SEMA5A/SEMA6D/SEMA6B/SEMA3E |
| neuropilin binding | GO:0038191 | 5.14E-06 | 0.00147 | 5 | MF | SEMA3F/SEMA3A/SEMA5A/SEMA3D/SEMA3E |
| transcriptional activator activity, RNA polymerase II transcription regulatory region sequence-specific binding | GO:0001228 | 4.30E-05 | 0.008199 | 24 | MF | FOXC1/DLX3/PITX1/TP63/FOSL2/TP73/GRHL2/DLX5/NR2E1/  EGR1/MEIS2/GRHL1/MYCN/TFAP2A/CREB3L4/MEIS1/HMGA2/  FOXH1/ATF3/PITX2/STAT6/ATF5/MAFF/ARID3C |

**Table 3. 5 μM PBB153**

| Description | ID | pvalue | padj | Count | Category | geneID |
| --- | --- | --- | --- | --- | --- | --- |
| thyroid hormone generation | GO:0006590 | 0.001931 | 0.014477 | 1 | BP | DIO2 |
| thyroid hormone metabolic process | GO:0042403 | 0.002413 | 0.014477 | 1 | BP | DIO2 |
| hormone biosynthetic process | GO:0042446 | 0.007831 | 0.031325 | 1 | BP | DIO2 |
| phenol-containing compound metabolic process | GO:0018958 | 0.011555 | 0.034207 | 1 | BP | DIO2 |
| translational elongation | GO:0006414 | 0.015033 | 0.034207 | 1 | BP | DIO2 |
| cellular modified amino acid metabolic process | GO:0006575 | 0.021731 | 0.034207 | 1 | BP | DIO2 |
| translational initiation | GO:0006413 | 0.022805 | 0.034207 | 1 | BP | EIF3CL |
| hormone metabolic process | GO:0042445 | 0.022805 | 0.034207 | 1 | BP | DIO2 |
| eukaryotic translation initiation factor 3 complex | GO:0005852 | 0.001936 | 0.001936 | 1 | CC | EIF3CL |
| translation initiation factor binding | GO:0031369 | 0.003742 | 0.015081 | 1 | MF | EIF3CL |
| translation initiation factor activity | GO:0003743 | 0.006032 | 0.015081 | 1 | MF | EIF3CL |
| translation factor activity, RNA binding | GO:0008135 | 0.010124 | 0.016873 | 1 | MF | EIF3CL |
| ubiquitin protein ligase binding | GO:0031625 | 0.035089 | 0.036632 | 1 | MF | DIO2 |
| ubiquitin-like protein ligase binding | GO:0044389 | 0.036632 | 0.036632 | 1 | MF | DIO2 |
